# Environmental and practice factors associated with children’s device-measured physical activity and sedentary time in early childhood education and care centres: a systematic review

**DOI:** 10.1186/s12966-022-01303-2

**Published:** 2022-07-14

**Authors:** Anne Martin, Rachel Brophy, Joanne Clarke, Charlotte J. S. Hall, Russell Jago, Ruth Kipping, Tom Reid, Benjamin Rigby, Hilary Taylor, James White, Sharon A. Simpson

**Affiliations:** 1 MRC/CSO Social and Public Health Sciences Unit, University of Glasgow, 99 Barkley Street, Glasgow, G3 7HR UK; 2 Population Health Sciences, Bristol Medical School, University of Bristol, Canynge Hall, 39 Whatley Road, Bristol, BS8 2PS UK; 3 Institute of Applied Health Research, University of Birmingham, Edgbaston, Birmingham, B15 2TT UK; 4 Centre for Exercise, Nutrition & Health Sciences, School for Policy Studies, University of Bristol, 8 Priory Road, Bristol, BS8 1TZ UK; 5 DECIPHer, Centre for Trials Research, University of Cardiff, Heath Park, Cardiff, CF14 4YS UK

**Keywords:** Physical activity, Sedentary behavior, Toddler, Preschooler, Early childhood, Centre-based childcare, Accelerometry, Outdoor, Indoor

## Abstract

**Background:**

Early childhood education and care (ECEC) settings offer a potentially cost-effective and sustainable solution for ensuring children have opportunities to meet physical activity (PA) and sedentary time (ST) guidelines. This paper systematically reviewed the association between childcare environment and practice and children’s PA and ST.

**Methods:**

Three electronic databases were searched, and citation tracking of eligible studies performed between June–July 2020 (updated March 2022). Studies were eligible when (i) participants attended ECEC settings, (ii) they reported the association between use of outdoor space, including factors of time, availability, play, size and equipment, and children’s device-measured PA and ST, and (iii) where applicable, they compared the exposure to use of indoor space. Risk of bias was assessed using the Critical Appraisal Skills Program (CASP) tools. A synthesis was performed using effect direct plots and charts to visualise effect sizes.

**Results:**

Of 1617 reports screened, 29 studies met the inclusion criteria. Studies provided data on outdoor versus indoor time (*n* = 9; 960 children), outdoor versus indoor play (*n* = 3; 1104 children), outdoor play space (*n* = 19; 9596 children), outdoor space use external to ECEC (*n* = 2; 1148 children), and portable (*n* = 7; 2408 children) and fixed (*n* = 7; 2451 children) outdoor equipment. Time spent outdoors versus indoors was associated with increased moderate-to-vigorous PA (MVPA), light PA (LPA) and total PA, while the association with ST was inconclusive. The mean (standard deviation) levels of outdoor MVPA (4.0 ± 3.2 to 18.6 ± 5.6 min/h) and LPA (9.9 ± 2.6 to 30.8 ± 11.8 min/h) were low, and ST high (30.0 ± 6.5 to 46.1 ± 4.3 min/h). MVPA levels doubled when children played outdoors versus indoors. Outdoor play space, and outdoor portable equipment, were associated with increased MVPA. A dose-response relationship for outdoor play area size was observed, demonstrating increased MVPA with areas ≥505m^2^ (5436 ft^2^), but no further increases when areas were > 900m^2^ (9688 ft^2^). No studies reported on injuries in outdoor settings.

**Conclusions:**

ECEC policies and practices should promote not only outdoor time but also the availability of resources such as portable play equipment and sufficient size of outdoor play areas that enable children to be physically active for sustained periods while outdoors.

**Systematic review registration:**

International prospective register of systematic reviews (PROSPERO) Registration Number: CRD42020189886.

**Supplementary Information:**

The online version contains supplementary material available at 10.1186/s12966-022-01303-2.

## Background

Review evidence shows that adiposity levels of young children aged 2–7 years were lower in children who engaged in more accelerometer-derived vigorous intensity physical activity (VPA) and moderate-to-vigorous intensity physical activity (MVPA) [1]. Total physical activity (TPA), MVPA and VPA benefit cognitive, motor, and socio-emotional development, as well as cardiometabolic health and sleep of young children [2, 3]. In contrast, spending extended periods of time sedentary in non-interactive activities has harmful effects on child health and development [3, 4]. In 2019, the World Health Organization released the 24-hour movement guidelines for children under the age of 5 years [5]. It is recommended that young children aged 3–4 years should be physically active for 180 minutes per day and sit for no more than an hour at a time. Of the 180 minutes per day of PA, children aged 3–4 years should spend 60 minutes per day in MVPA. For older children (i.e. those aged 5 to 17 years), the World Health Organization recommends engagement in an average of at least 60 minutes MVPA per day across the week [6]. Failure to meet the recommended amount of physical activity (PA) in early childhood has been shown to track into adolescence [7] and across the lifespan [8, 9].

Early childhood education and care (ECEC) settings present a unique opportunity for promoting PA during weekdays through structured exercise or active play [10, 11]. Many children attend ECEC settings. For example, the average enrolment rate is 87% for 3–5 year olds in OECD countries [12], highlighting that educational settings offer a potentially cost-effective, replicable and sustainable solution to ensuring that children are provided with opportunities to be active.

A 2018 systematic review of the international literature (55 studies) from 11 countries indicated that accelerometer-derived PA levels and sedentary time (ST) of preschoolers aged 2–5 years differed widely with TPA ranging on average from 4 to 47 min/h, MVPA from 1 to 23 min/h and ST from 12 to 56 min/h during ECEC attendance [13]. The wide range of estimates might be a product of different geographical contexts and accelerometer cut-offs used to determine PA intensity, and combining of outdoor and indoor PA. Several studies have explored the difference in outdoor time at ECEC compared to indoor time on PA and ST in young children. One study found that children spent significantly less time sedentary (51% of time compared to 75%) and a greater amount of time in MVPA (31% of time compared to 12%) when outdoors in comparison to indoors [14]. This is consistent with other studies that have found children to be more active when outdoors in ECEC settings [15, 16]. However, other research suggested that MVPA levels are lower and ST higher outdoors, while only LPA is higher outdoors compared to indoors at ECEC [17]. Systematic review evidence of how active children are during outdoor playtime revealed that 14% of outdoor playtime was spent in MVPA, 44% in TPA and 53% sedentary [18]. This suggests that ST is high, and time spent in higher intensity physical activity is low.

Research has explored the factors involved in enabling children to be physically active during their time at ECEC including PA policies and educators’ active involvement. While the presence of PA policies at ECECs (e.g. the WHO standards for healthy eating and movement behaviours in ECEC settings [19]) and educators’ active involvement in PA have been related to increased PA levels of preschoolers [20–22], research has shown that the physical environment influences children’s PA levels. According to the theory of affordances, there is an interaction between what the environment offers the child, children’s perception of the environment and children’s intentions, previous experiences and the context [23]. Research on affordances of the ECEC environment found that both physical (e.g. terrain, vegetation) and social (e.g. educators and other children) affordances are associated with children’s physical activity levels [24]. Tonge et al.*’*s systematic review of correlates of children’s objectively measured PA and ST in ECEC, suggested that presence of an outdoor space in childcare and the size of the play area were amongst the most strongly associated factors impacting children’s levels of PA [25]. Dowda et al. [26] also found that a larger playground area was significantly associated with less ST and more MVPA for preschoolers. Conversely, other research indicated no association between children’s MVPA and the size of the outdoor play space [17]. The same study also found that the presence of portable and fixed equipment did not have a significant impact on MVPA [17]. However, the findings of a natural experimental study indicated that an upgrade in outdoor portable play equipment had a significantly positive impact on MVPA levels of pre-schoolers aged 2–5 years [27]. The inconsistency of findings might be a function of the complexity of the child-environment relationship and ability to attribute characteristics of the outdoor environment of ECEC settings to child PA and ST.

Whist previous reviews have examined the potential benefits of outdoor versus indoor PA, none have examined the association with accidents and injuries. A single study involving 2105 Norwegian ECECs indicated that most injuries, which were typically minor and more common in boys, occur outdoors [28]. However, the associations between outdoor PA and play and injury are unclear. Past research has largely examined *risky play*, which involves experimenting with uncertainty and overcoming fears, and is more common outdoors [29]. Due to the concerns among ECEC staff [30, 31] and wider societal pressures including the fear of litigation [31, 32], various injury prevention strategies are often imposed in ECEC on children’s outdoor PA. These include education campaigns [33], regulatory environmental changes (e.g. equipment) [34], and limitations on outdoor use and play (e.g. remaining indoors during rain or banning climbing) [30]. However, the unintended consequence of such strategies may be reduced PA and play. To help avoid ECEC injury prevention strategies that preclude healthy child development, especially PA promotion, and heed calls to examine the risk-benefit trade-off of outdoor versus indoor PA and play [32], an evidence synthesis examining the incidence rate, severity and type of injuries is required.

A systematic review of the literature and synthesis of findings is warranted to explore if recommendations for research, policy and practice can be made to support population health initiatives for child physical activity at ECEC. To-date, there has been no attempt to synthesise the literature to summarise associations on the influence of outdoor versus indoor ECEC environments on children’s PA and ST, and the complexities of the child environment association.

Therefore, the aim of this study was threefold: to systematically review and synthesise the published evidence (1) investigating how much PA children obtain, and how much time they spend sedentary, outdoors compared to indoors while attending ECEC, and how PA patterns differ by PA intensity and ST; (2) assessing the influence of the physical environment and practices on children’s PA and ST; and (3) addressing if there are more or different types of injury in young children during outdoor PA compared to indoor PA while attending ECEC.

## Methods

This systematic review was performed in accordance with the Preferred Items for Systematic Reviews and Meta-analyses (PRISMA) statement [35]. It was prospectively registered with PROSPERO (registration number: CRD42020189886). Deviations from the protocol are justified in Additional file 1.

### Eligibility criteria

The following criteria were applied to determine studies eligible for inclusion:

#### Population

Children with a mean age between 2 and 7 years, without diagnosed acute or chronic health conditions, attending ECECs (full or part-time), and who were not eligible for transition to primary or elementary school education.

#### Exposure(s)

Use of outdoor space including, but not limited to, the factors of time, availability (yes/no), play, size and portable and fixed outdoor play equipment.

#### Comparator(s)

Where applicable, the use of indoor space including, but not limited to, the factors of time and play.

#### Outcome(s)

Device-measured time spent in PA (TPA, MVPA, VPA, light intensity PA (LPA) and step counts); device-measured ST; and injuries (including number, type, and severity). Accelerometers have the ability to capture different PA intensities in short timeframes and over multiple planes that direct observations are not able to do. Device-based assessment also allows objective understanding of daily PA and ST which would be impractical to do with direct observations and would require repeated measures which may induce reactivity effects leading to a change in usual behaviour [36].

#### Study designs

Cohort studies (cross-sectional and longitudinal) irrespective of whether outcomes were assessed in the same child both indoors and outdoors, case-control studies (i.e. non-randomised controlled before and after studies), or (cluster) randomised controlled trials (RCTs).

#### Setting

Nursery school, preschool, kindergarten, and childcare centres in high-income countries, as defined by the World Bank [37].

#### Follow-up

Any or no follow-up period.

#### Report characteristics

Peer-reviewed scientific journal articles of primary research published in English since 1997. The language criterion was applied for feasibility, while the publication date represents when accelerometers became available to measure PA levels. Systematic reviews, other literature reviews, conference abstracts and unpublished manuscripts were excluded.

### Search strategy and selection process

Three electronic databases (MEDLINE within Ovid, and PsycINFO and SPORTDiscus within EBSCOhost) were searched on 4th and 5th June 2020. Forward and backward citation searching of eligible studies was conducted on 16th July 2020, using Google Scholar and Web of Science (Clarivate), respectively. An update literature search was conducted on 25th March 2022 (MEDLINE and PsychINFO) and 31st March 2022 (SPORTDiscus). The search strategy was informed by previous reviews [18, 38]. It included subject headings and keywords relating to the population (i.e. young children), exposure and comparator (i.e. use of outdoor and indoor space), setting (i.e. early childhood education centres), and outcome (i.e. device-based PA or ST assessment). Additional file 2 provides the line-by-line search strategy run in all three databases.

Identified records were exported to EndnoteX9 [39] and uploaded to Covidence systematic reviewing software [40] for deduplication and study selection. The selection process was piloted among reviewers on a sample of eight records to ensure consistency.

Identified records were screened once for eligibility (a 10% sample were independently double-screened), initially by title and abstract (CJSH, HT, RB, TR), before full-text screening was conducted on potentially relevant articles (50% independently double-screened; CJSH, HT, RB, TR). Discrepancies between reviewers at both stages were resolved by a third reviewer (AM). Records were excluded if full texts were irretrievable, or where insufficient information precluded eligibility assessment. For pragmatic reasons, publication authors were not contacted.

### Data collection

Data were extracted (CJSH, TR, JC) using a data extraction form, which was piloted on two full-text articles, and independently crossed-checked by a second reviewer (AM, RB). Disagreements were resolved through discussion.

The following outcomes data were collected: description (i.e. PA, PA intensity, steps, ST); measurement (i.e. assessment tool, including model of accelerometer or pedometer); units (e.g. min/hr); method of data processing; accelerometer cut-points for classifying the intensity of PA or ST; epochs (i.e. the usual accelerometer stored magnitude of accelerations at fixed recording intervals); and the number of time points, attrition and missing data.

Additional data extracted were: publication details (authors, year, study design, country); population (sample size, age [mean, SD/SE, range], gender); exposure (description of availability and number of outdoor play spaces, number and types of fixed and/or portable outdoor play equipment, use of outdoor space external to ECEC setting, outdoor play intervention, outdoor play time, assessment tools of exposure [including units where applicable], duration, frequency); comparison condition (description of the availability and number of indoor play/physical activity space and dedicated indoor play time, duration, frequency); and results at baseline and, where applicable, follow-ups (effect estimates and CIs [SD/SE] and/or mean/median [SD/SE] for each time point and/or effect direction).

### Study risk of bias

Modified versions of the Critical Appraisal Skill Program (CASP) tools for RCTs, case control studies and cohort studies were used to assess risk of bias in eligible studies. The modifications can be found in Additional file 3. The different tools, which apply to specific study designs, prompted reviewers to consider each study’s design validity and quality of results [41–43]. The tools cover domains including acceptability of recruitment, measurement of exposure and outcome, accounting for confounding factors, adequate follow-up, and precision and trustworthiness of results.

All studies were independently assessed in duplicate (TR and AM, HT and AM, HT and RB, or RB and AM). Reviewers selected a response of ‘yes’, ‘can’t tell’ or ‘no’ against each item. Discrepancies were resolved through discussion. Studies with > 50% of ‘yes’ responses were deemed to be at low risk of bias.

### Synthesis methods

Data were unsuitable for the planned within-subject multivariate meta-analysis, for several reasons: i) uncertainty of covariance between PA levels indoors and outdoors; ii) incomparable exposure measurements (outdoor play area size); and iii) too few studies (remaining exposures). Therefore, narrative synthesis was adopted in-line with Synthesis Without Meta-analysis (SWiM) guidance [44].

Studies were grouped by exposure and then by outcome. Where possible (i.e. for MVPA for the outdoor versus indoor play comparison, and MVPA and ST for the exposure outdoor play area size), mean differences were calculated, and the interpretation of effects was based on confidence intervals if reported. Data were further prepared for synthesis by calculating means and SDs as appropriate. When necessary, standard deviations were calculated from standard error statistics and confidence intervals or estimated from *p*-values and between group t-statistics. Where data were unsuitable for conversion, the units of measures reported by the study authors were used.

Effect direction plots were generated for each exposure to ascertain if there was any evidence of effect for each outcome [45]. Data were suitable for inclusion in plots whereby two or more studies examined the same outcome, irrespective of measurement units. The plots visualised the study design, sample size, risk of bias and effect direction for each study, and provided an overall summary effect direction across the studies for each outcome. Bar charts were created for the exposures outdoor time and outdoor play area size, depicting min/h in ECEC for MVPA, ST and LPA (outdoor time only), which were converted from reported data.

Where data allowed, results were presented indicating different accelerometer cut-points used by the study authors and child gender. The proposed subgroup analyses by follow-up duration and studies with within-person outcome assessment were not performed due to the high proportion of cross-sectional designs examining within-person comparisons of indoor versus outdoor.

### Reporting bias assessment

Publication bias assessment was planned but not performed as fewer than 10 studies assessed any single outcome [46].

## Results

### Study selection

**Fig. 1:**
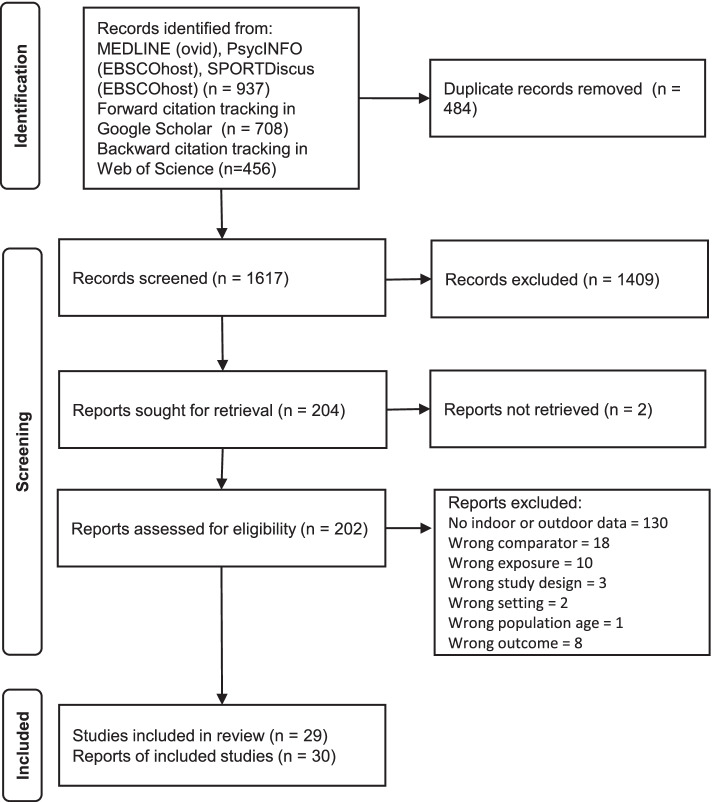
Flow diagram of literature search

The literature search identified 2101 records. Four hundred eighty-four duplicates were excluded before screening. One thousand six hundred seventeen records were screened by title and abstract, of which 1409 were excluded. 204 reports were retrieved for detailed evaluation, while 2 potentially eligible reports were irretrievable. One hundred seventy-two retrieved reports did not meet inclusion criteria, resulting in 30 reports (29 studies) being included in this review. Figure 1 shows the flow of studies during the literature search and reasons for the exclusion of records deemed ineligible for review.

### Study characteristics

Studies examined different types of outdoor exposure at ECECs with children’s PA and ST. Therefore, studies were grouped into at least one of seven different types of outdoor exposure categories: outdoor versus indoor time, engaging in outdoor play, outdoor play space, use of outdoor space external to ECEC premises, and outdoor play equipment. Tables 1, 2, 3, 4 and 5 in Additional file 4, summarise the study characteristics for each exposure category.

### Outdoor versus indoor time

Ten studies compared children’s PA and ST during time spent outdoors versus indoors (Table 1 in Additional file 4). All but one study were cross-sectional; Trost et al. conducted a randomised controlled trial [47]. Five studies were conducted in the USA [14, 17, 47–49] and one each in Canada [50], Spain [51] and Norway [52]. A further study compared data across both the USA and Sweden [16]. The sample sizes ranged from 31 children across 13 ECECs [50] to 388 children across 30 ECECs [17]. Outdoor and indoor time was recorded by ECEC staff in one study by rating per hour on a four-point scale [52]. The Observational System for Recording Activity in Preschoolers (OSRA-P) tool was used by researchers in one study [47], and the remaining five did not specify the tools used, typically reporting that researchers recorded observations of the children [16, 17, 49, 50]. One study recorded time by video recording [48] and one with QStarz GPS devices [14, 17, 47–52].

### Engaging in outdoor versus indoor play

Three studies assessed the association between engaging in outdoor play, and PA and ST (Table 2 in Additional file 4). All studies were conducted in the USA and were cross-sectional in design. The sample sizes ranged from 98 children across 10 ECECs [49] to 559 children across 50 ECECs [53]. Direct observation noting child location and if they were engaging in active outdoor play was employed in one study [49]. The Environment and Policy assessment and Observation (EPAO) tool was utilised in one study [53]. Another one used an environmental audit developed by the study authors [54].

### Outdoor play space

Three different exposures relating to the outdoor play space were studied and examined in relation to PA and ST (Table 3 in Additional file 4).

#### Availability of outdoor play area

Four cross-sectional studies conducted in the Netherlands [55], Denmark [56], the USA [57] and Canada [58] assessed children’s PA levels and ST between the availability of an outdoor play area with availability of an indoor play area at ECECs. All studies used direct observations, each with slightly different measures of outdoor and indoor play area. Gubbels et al. compared the number of indoor and outdoor active play areas [55]. Olesen et al. assessed the number of ECEC sides that were accessible for the children when playing on the playground and the number of rooms for children to be active daily [56]. Meanwhile, Zhang et al. assessed the functional and developmental needs of playground [58] and Stephens et al. assessed the availability of outdoor and indoor play space [57].

#### Absolute size of the outdoor play area

Eleven studies (12 articles) related the size of the outdoor play area to children’s PA and ST. Of the 11 studies, nine were cross-sectional studies, one was a controlled before-after study [27] and one was a randomised controlled trial [59]. Five studies were conducted in Australia [20, 27, 60–62], three in the USA [26, 54, 59] and one in Denmark [56]. One study, reported in two articles, was conducted in both the USA and Sweden [16, 63]. Sample sizes ranged from 107 children across 10 ECECs [61] to 1002 children across 136 ECECs [60]. Three studies compared movement behaviours of outdoor play areas ≤400m^2^ versus >400m^2^ [20, 61, 62]. Two studies used smaller sizes of outdoor play areas as reference thresholds for comparison: ≤200m^2^ versus 900m^2^ and > 2700 m^2^ [64], and < 386 m^2^ versus ≥386m^2^ [26]. Another three studies defined larger playground sizes for comparison: <505m^2^ versus ≥505m^2^ [54], < 1038 m^2^ versus ≥1038m^2^ [59], and < 1200 m^2^ versus 3000m^2^ and ≥ 3000 m^2^ [16, 63]. Olesen et al. measured the size of accessible playground area across ECECs and used them as continuous variables ranging from 567m^2^ to 5175 m^2^ [56]. Two studies measured but did not report the size of the playground [27, 60].

#### Density of the outdoor play area

Two studies assessed the association between the size of the outdoor play area per child (m^2^ per child) and children’s PA and ST. [55, 65] One was a Belgian before-and-after study that examined changes in PA after reducing the number of ECEC classes sharing the playground during recess time, which led to an increase in space per child from 7.4m^2^ to 16.7m^2^ [65]. This study included 128 children across 22 ECECs. Gubbels et al.*’*s cross-sectional study compared the relative size of outdoor free play space (mean m^2^ per child = 42.9 ± 45.6) for 152 children across 22 ECECs in the Netherlands [55]. Similarly, another three studies of cross-sectional design from Belgium [66] and Spain [51, 67], related the average number of children per m^2^ with PA of 789 children across 39 ECECs [66], 116 children across six ECECs [51], and 120 children across seven ECECs [67].

### Use of outdoor space external to ECEC premises

One cross-sectional study conducted in Finland assessed the association between frequency of nature visits and frequency of visits to play parks with ST in 778 children across 66 preschools [68]. Another study conducted in Brazil in 370 children across 8 preschools related the availability of a nearby park with children’s PA and ST. [69] In both studies, information about the exposure were collected via questionnaire completed by educators (Table 4 in Additional file 4).

### Outdoor play equipment

Two different exposures relating to the outdoor play equipment were studied and examined in relation to PA and ST (Table 5 in Additional file 4).

#### Portable outdoor play equipment

Seven studies assessed the association between portable outdoor play equipment and children’s PA and/or ST in ECECs [17, 26, 27, 55, 56, 68, 70]. While two cross-sectional studies [17, 55] compared the number of pieces of outdoor and indoor portable equipment and four studies (three cross-sectional, one controlled pre-post study) assessed availability of portable outdoor play equipment [26, 27, 56, 68], Hannon and Brown (2008) introduced activity-friendly play equipment outdoors as part of an intervention, and compared children’s activity levels before and after providing the play equipment [70]. Three studies were conducted in the USA with a sample size of 388 children (30 ECECs) [17], 299 children (24 ECECs) [26], and 64 children (1 ECEC) [70]. One study each was conducted in Finland with 778 children (66 ECECs) [68], Denmark with 441 children (42 ECECs) [56], The Netherlands with 152 children across 22 ECECs) [55], and Australia with 297 children (11 ECECs) [27].

#### Fixed outdoor play equipment

Seven studies assessed the association between fixed outdoor play equipment and children’s PA and/or ST. [17, 26, 27, 55, 56, 61, 68] Of these, six studies assessed also portable outdoor play equipment [17, 26, 27, 55, 56, 68]. The additional study was cross-sectional in design and conducted in Australia with 107 children (10 ECECs) [61].

### Outcome assessment

All studies used accelerometers to measure PA, with the exception of three studies that used pedometers [20, 63, 66]. Four different models of ActiGraph accelerometers were used in the included studies: 7164 [26, 47], GT1M [16, 48, 52–54, 56, 59, 61, 65, 70], WGT3XBT [58], and GT3X [14, 27, 49, 52, 55–57, 59, 62, 64, 68, 69]. ActiCal accelerometers were used in two studies [17, 50]. All studies used a 15 s epoch length, except for two studies using a 10s epoch [55, 64], one study using a 5 s epoch [54], and one study using a 1 s epoch [69]. Various cut-points were used (Table 6 in Additional file 4).

Eleven studies measured TPA [16, 27, 48, 50, 52, 53, 60, 62, 68, 69]; 24 studies measured MVPA [14, 16, 17, 26, 27, 47, 49, 50, 53–57, 59, 61, 62, 64, 65, 69, 70]; 10 studies measured LPA [14, 16, 17, 49, 53, 64, 70]; two studies measured VPA [51, 70]; one study measured light-to-vigorous intensity physical activity (LMVPA) [65]; and four studies measured step count [20, 63, 64, 66]. Sedentary time, also expressed as sedentary behaviour or activity, was measured in 17 studies [14, 16, 17, 26, 48–50, 53, 55, 61, 62, 64, 65, 68–70]. No study reported injuries related to outdoor experiences at ECEC.

### Risk of bias in studies

Thirty published reports including 25 cross-sectional studies, four RCTs and one case-control study (CCS) were assessed for quality using modified versions of the CASP tools (Additional file 3). No study met all eight quality assessment criteria (nine for the CCS report; or six in two RCTs whereby two criteria were irrelevant). Overall, 11 studies were rated as having a low risk of bias (cross-sectional = 10 [20, 26, 50, 52, 54–57, 68, 69]; RCT = 1 [47]).

Participants were recruited through random sampling in the CCS and 12 cross-sectional studies, however 12 of the cross-sectional studies reported insufficient information to assess the recruitment methods. Most studies took account of confounding factors in their design and/or analysis, but only nine were considered to have identified important confounding factors [17, 50, 54–57, 61, 63]. Commonly missed factors included accelerometer wear time, child age, clustering of children in childcare centres and socioeconomic status.

Sixteen cross-sectional studies were deemed to have minimised bias in outcome measurement [14, 16, 20, 26, 50, 51. 52, 53, 56, 58, 62, 63, 64, 66, 68, 69], but only 8 did so for exposure measurement [9, 14, 48, 50, 53–55, 58]. All remaining but one [52] cross-sectional studies, and the CSS [27], received *‘can’t tell’* ratings for efforts to minimise bias in exposure measurement. Commonly, this was due to insufficient detail about methods used to record exposures. All four RCTs were rated as *‘can’t tell’* for whether the outcome had been accurately assessed to minimise bias [47, 59, 65, 70].

### Synthesis of results

#### Comparison of outdoor and indoor time

##### Physical activity

**Fig. 2:**
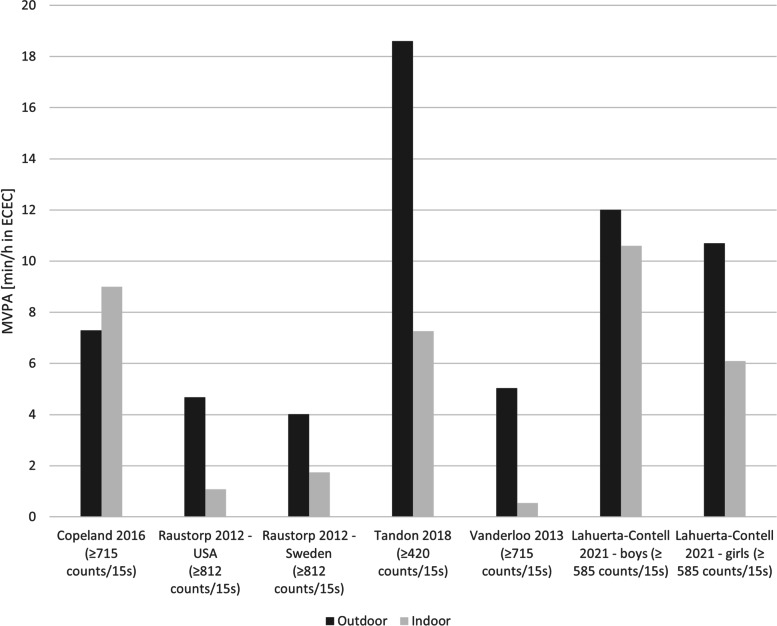
Accumulation of moderate-to-vigorous intensity physical activity (MVPA) compared between outdoor and indoor time. In brackets are the used accelerometer cut-off points

**Fig. 3:**
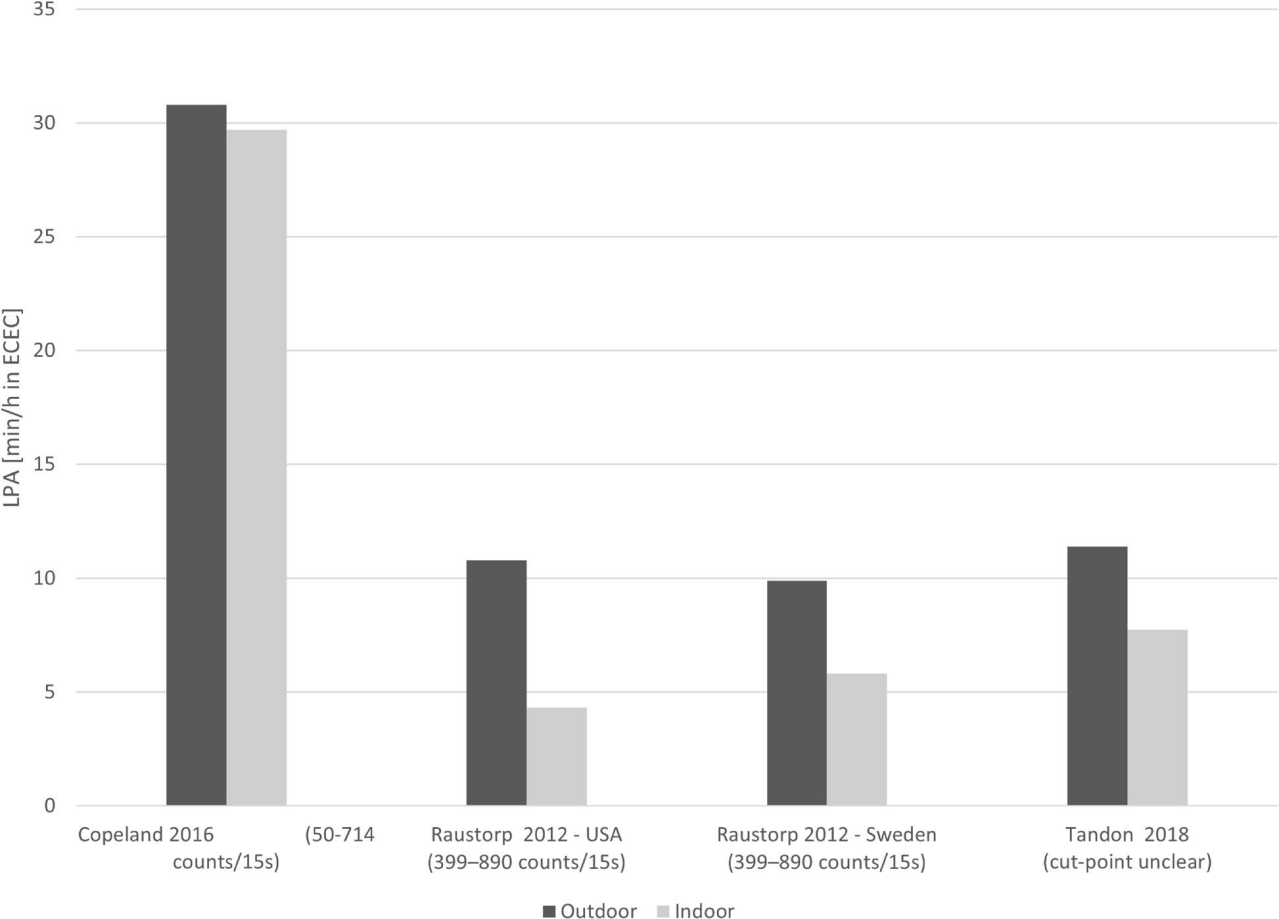
Accumulation of light intensity physical activity (LPA) compared between outdoor and indoor time. In brackets are the used accelerometer cut-off points

Nine studies compared children’s PA levels during time spent outdoors versus indoors. Table 1 summarises the effect directions and indicates that children accumulated more LPA, MVPA and TPA during outdoor time compared to indoor time. However, time spent in MVPA during outdoor time ranged on average between 4.0 (SD 3.2) min/h to 18.6 (SD 5.6) min/h while attending ECEC settings (Fig. 2). Similarly, for studies where suitable data were available, Fig. 3 shows the accumulation of LPA during ECEC time with outdoor LPA levels ranging from 9.9 (SD 2.6) min/h to 30.8 (SD 11.8) min/h. Five studies (5 cohorts) investigated gender differences and found that in 4/5 cohorts, boys were more physically active outdoors (MVPA and TPA) than girls [14, 16, 50, 51] and that the difference between PA levels outdoor versus indoors were bigger for boys [14, 16, 50]. In contrast, data of children in Sweden suggested that girls were more active outdoors than boys [16] and in a Norwegian cohort, girls were equally as active outdoors as boys [52].

**Table 1:** Comparison of physical activity levels and sedentary time spent outdoors relative to indoors in ECEC

Study ID	Study Design	Sample size	Risk of bias	Sedentary time	LPA	MVPA	Total PA
Andersen 2017 [52]	Cross-sectional	116	Low	**–**	**–**	**–**	▲
Copeland 2016 [17]	Cross-sectional	388	High	▼	▲	▼	**–**
Lahuerta-Contell 2021 [51]	Cross-sectional	116	High	–	–	▲	**–**
Raustorp 2012 [16]	Cross-sectional	50	High	▲	▲	▲	▲
Schlechter 2017 [48]	Cross-sectional	73	High	▲	**–**	**–**	▲
Tandon 2015 [49]	Cross-sectional	98	High	▲	**–**	▲	**–**
Tandon 2018 [14] {Tandon, 2018 #24}	Cross-sectional	46	High	▲	▲	▲	**◄►**
Trost 2008^a^ [47]	RCT	20	Low	**–**	**–**	▲	**–**
Trost 2008^b^ [47]	RCT	22	Low	**–**	**–**	▲	**–**
Vanderloo 2013 [50]	Cross-sectional	31	High	▲	**–**	▲	**–**
**Summary effect direction**				**◄►**	▲	▲	▲

*Abbreviations*: *LPA* Light intensity physical activity, *MVPA* Moderate-to-vigorous physical activity, *TPA* Total physical activity; ^a^ = intervention group; ^b^ = control group

Effect direction: Study level: ▲ = outdoor time benefits outcomes (lower sedentary time; higher physical activity); ▼ = outdoor time not associated with improvements in outcomes (higher sedentary time; lower physical activity); ◄► = conflicting findings; ‘-‘= outcome not assessed

Summary: ▲ = studies show a positive association with outdoor time at ECEC; ◄► = conflicting findings

##### Sedentary time

**Fig. 4:**
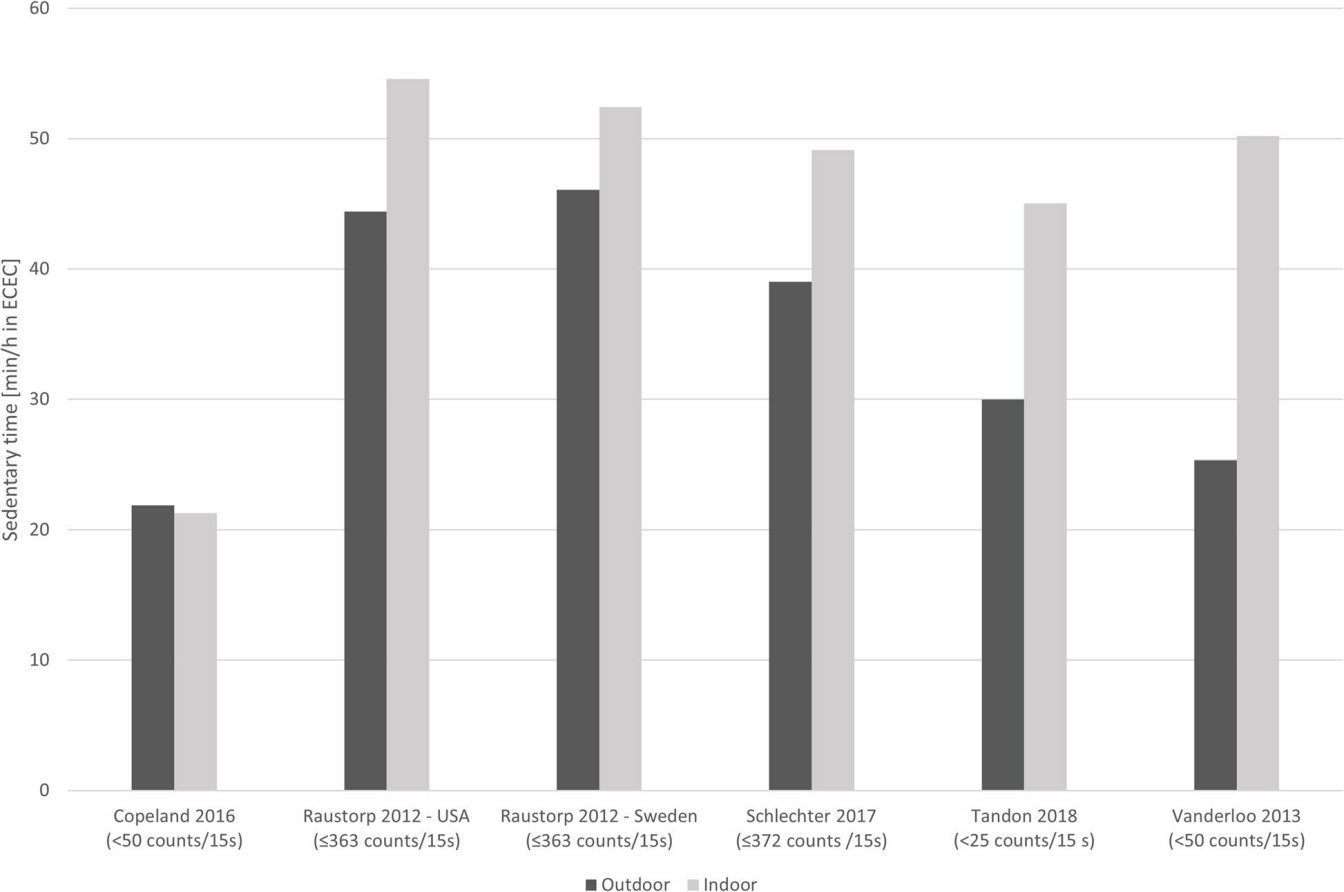
Comparison of time spent sedentary during outdoor versus indoor time during ECEC attendance. In brackets are the used accelerometer cut-off points

Six studies assessed the difference in ST during outdoor and indoor time in ECECs. The summary effect direction in Table 1 suggests conflicting findings which could be explained by 1-day accelerometer wear time protocol of the largest study [17]. While five out of six studies indicated that children spend less time sedentary when being outdoors, the accumulated ST outdoors ranged from 30.0 (SD 6.5) min/h to 46.1 (SD 4.3) min/h (Fig. 4). Two studies provided data for boys and girls separately [16, 50]. Data suggested that girls were more sedentary outdoors than boys and that the difference in outdoors versus indoors sedentary time was bigger for boys.

#### Engaging in outdoor versus indoor play

##### Physical activity

Table 2 shows that both studies that compared accumulated LPA and MVPA during outdoor play and indoor play sessions observed higher PA levels when children played outdoors. Mazzucca et al. suggested that children spent 20.9 min/h in LPA outdoors compared to 17.2 min/h indoors [53]. MVPA levels doubled when children played outdoors but accumulated time was low in both studies with an outdoor-to-indoor mean difference of 7.6 min/h and 1.0 min/h in Mazzucca et al. [53] and Tandon et al. [49], respectively. Henderson et al. measured MVPA during outdoor and indoor play time in relation as to whether educators encouraged or participated in play [54]. Effect directions suggested that when educators did not encourage PA during play, children spent more time in MVPA outdoors compared to indoors (1.2% of wear time), whereas educator encouragement led to less time spend in MVPA outdoors than indoors (− 1.8% of wear time) [54]. Only one study explored gender differences and found an effect direction with higher LPA and MVPA in boys compared to girls [49].

**Table 2:** Comparison of outdoor with indoor ECEC play on LPA, MVPA and sedentary time

Study ID	Study Design	Sample size	Risk of bias	Sedentary time	LPA	MVPA
Henderson 2015 [54]	Cross-sectional	447	Low	–	–	**◄►**
Mazzucca 2018 [53]	Cross-sectional	559	High	▲	▲	▲
Tandon 2015 [49]	Cross-sectional	98	High	▼	▲	▲
**Summary effect direction**				**◄►**	▲	▲

*Abbreviations*: *LPA* Light intensity physical activity, *MVPA* Moderate-to-vigorous physical activity

Effect direction: Study level: ▲ = outdoor play benefits outcomes (lower sedentary time; higher physical activity); ▼ = outdoor play not associated with improvements in outcomes (higher sedentary time; lower physical activity); ◄► = conflicting findings

Summary: ▲ = studies show a positive association with outdoor play at ECEC; ◄► = conflicting findings

##### Sedentary time

Comparison of time spent sedentary during outdoor and indoor play time revealed inconsistent results (Table 2). While Mazzucca et al. suggested lower ST during outdoor play time compared to indoor play time (23.5 vs 34.7 min/h) [53], Tandon et al. indicated 0.5 min/h more ST outdoors with boys being less sedentary outdoors than girls [49]. Inconsistency could not be explained by the different accelerometer cut-off used because both studies used < 25 counts/15 s as cut-off for sedentary time.

#### Association of outdoor play space on physical activity and sedentary time

##### Physical activity

Four studies assessed the association between *availability of outdoor play areas* and children’s MVPA levels. Overall, availability of dedicated outdoor play space was associated with increased levels of MVPA, with nearly 1 min/h [57] and 0.3% monitored time more [56], compared to ECECs without dedicated outdoor play space (Table 3). Gubbels et al. did not report any summary statistics on availability of outdoor play areas but indicated that there was no significant association [55]. Zhang et al. conducted a more detailed analysis into the functional and developmental needs of outdoor play space of toddlers (mean age 2.2 ± 0.4 yrs) and preschoolers (mean age 3.4 ± 0.6 yrs). Outdoor play space meeting both functional and developmental needs of preschoolers resulted in increased MVPA during ECEC time: B = 0.15 min/h (95% CI 0.05 to 0.25) and B = 0.14 min/h (95% CI 0.01 to 0.28), respectively [58]. For toddlers, functional and developmental needs were non-significantly associated with lower MVPA. Findings for LPA were similar for both preschoolers and toddlers. Outdoor play space meeting the functional and developmental needs of preschoolers showed an association with increased LPA levels: B = 0.62 min/h (95%CI − 0.71 to 1.95) and B = 2.35 min/h (95% CI 0.87 to 3.83), respectively [58].

**Table 3:** Availability of outdoor play space at ECEC on MVPA

Study ID	Study Design	Sample size	Risk of bias	MVPA
Gubbels 2018 [55]	Cross-sectional	281	Low	■
Olesen 2013 [56]	Cross-sectional	426	Low	▲
Stephens 2014 [57]	Cross-sectional	491	Low	▲
Zhang 2021^a^ [58]	Cross-sectional	242	High	▲
**Summary effect direction**				▲

*Abbreviations***:** *MVPA* Moderate-to-vigorous physical activity. ^a^ = preschoolers only

Effect direction: Study level: ▲ = availability of outdoor play space benefits outcomes (higher physical activity); ■ = (summary) statistics not presented

Summary: ▲ = studies show a positive association with availability of outdoor play space at ECEC

**Fig. 5:**
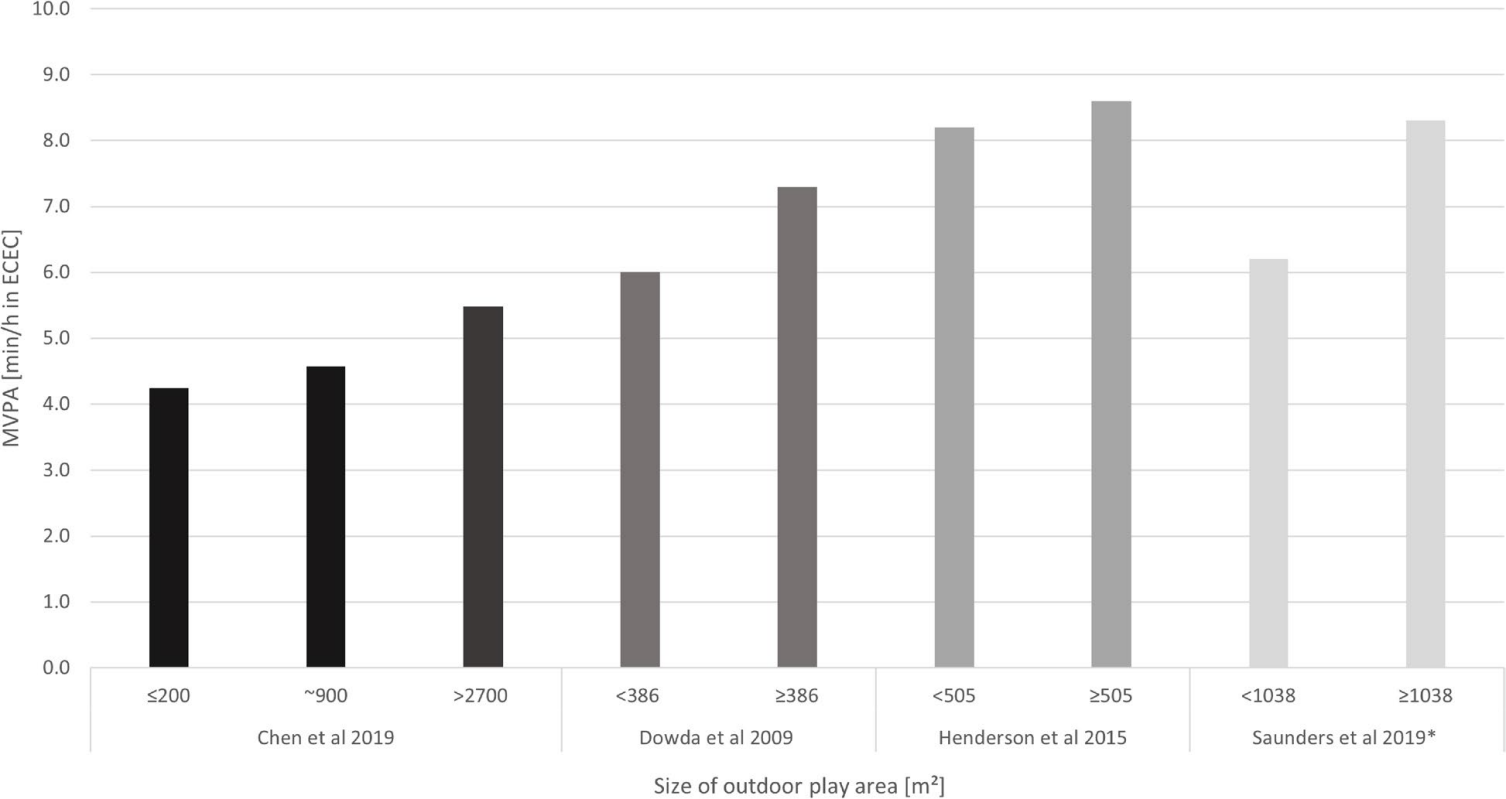
Moderate-to-vigorous intensity physical activity by size of outdoor play area. *denotes that control group data are displayed

Eleven studies assessed the association between the a*bsolute size of the outdoor play area* and PA levels. Synthesis of effect directions indicated that bigger outdoor play areas were associated with higher levels of MVPA and step counts compared to smaller outdoor play areas (Table 4). Figure 5 shows the accumulation of MVPA in minutes per hour ECEC time for compared sizes of outdoor play areas. Where data were available mean differences were calculated. The mean difference in MVPA between an outdoor play area < 386 m^2^ and ≥ 386 m^2^ was 1.30 min/h (95% CI − 1.15 to 3.75) [26], between <505m^2^ and ≥ 505 m^2^ was 0.38 min/h (95% CI 0.28 to 0.48) [54], and between ≤200m^2^ and > 2700 m^2^ was 1.24 min/h (95% CI 0.59 to 1.89) [64]. Olesen et al. considered the size of the outdoor area as continuous variable with a median size of 2700m^2^ and found no association with MVPA levels (0.0% monitored time, 95% CI 20.0 to 0.0) [56]. Only one study explored differential association for boys and girls [60] and findings suggested that the size of the outdoor area was associated with girls’ TPA but not boys’.

**Table 4:** Absolute size of outdoor play space at ECEC on physical activity and sedentary time

Study ID	Study Design	Sample size	Risk of bias	Outdoor play area size	Sedentary time	MVPA	TPA	Steps
Bell 2015 [20]	Cross-sectional	328	Low	≤ 400m^2^ vs. > 400m^2^	**–**	**–**	**–**	▲
Boldeman 2011 [63]	Cross-sectional	169	High	< 1200m^2^ vs. 1200-3000 m^2^ vs. >3000m^2^	**–**	**–**	**–**	■
Chen 2020^1^ [64]	Cross-sectional	69	High	≤200m^2^ vs. ~900m^2^	▲	▲	**–**	▲
Chen 2020^2^ [64]	Cross-sectional	151	High	≤200m^2^ vs. >2700m^2^	▲	▲	**–**	▲
Dowda 2009 [26]	Cross-sectional	299	Low	< 387m^2^ vs. ≥ 387m^2^	▲	▲	**–**	**–**
Henderson 2015 [54]	Cross-sectional	447	Low	< 505m^2^ vs. ≥ 505m^2^	**–**	▲	**–**	**–**
Hinkley 2016 [60]	Cross-sectional	731	High	Not reported	**–**	**–**	■	**–**
Ng 2020^a^ [27]	Case-control	120	High	Not reported	**–**	■	■	**–**
Ng 2020^b^ [27]	Case-control	103	High	Not reported	**–**	■	■	**–**
Olesen 2013 [56]	Cross-sectional	426	Low	567m^2^-5175m^2^, median 2700m^2^	**–**	◄►	–	**–**
Saunders 2019^a^ [59]	RCT	188	High	<1308m^2^ vs. ≥ 1308m^2^	**–**	▲	**–**	**–**
Saunders 2019^b^ [59]	RCT	191	High	<1308m^2^ vs. ≥ 1308m^2^	**–**	▲	**–**	**–**
Sugiyama 2012 [61]	Cross-sectional	107	High	≤ 400m^2^ vs. > 400m^2^	◄►	▲	**–**	**–**
Tonge 2020 [62]	Cross-sectional	490	High	< 400m^2^ vs. ≥ 400m^2^	▲	▲	▲	**–**
**Summary effect direction**					▲	▲	◘	▲

*Abbreviations*: *MVPA* Moderate-to-vigorous physical activity, *TPA* Total physical activity; ^a^ = intervention group; ^b^ = control group; ^1^ = first outdoor play area size comparison; ^2^ = second outdoor play area size comparison

Effect direction: Study level: ▲ = Absolute size of outdoor play space benefits outcomes (lower sedentary time; higher physical activity); ◄► = conflicting findings; ■ = (summary) statistics not presented; ‘-‘= outcome not assessed

Summary: ▲ = studies show absolute size of outdoor play space at ECEC benefits outcomes (lower sedentary time; higher physical activity); ◘ = insufficient reporting of data

Five studies assessed the association between the *density of the outdoor play area* and PA. Reducing the number of ECEC classes sharing the playground during recess time, which led to an increase in space per child from 7.4m^2^ to 16.7m^2^, was associated with 0.8 min (4.7% of recess time) increase in MVPA and 1 min (5.1% of recess time) in TPA [65]. A study assessing the average number of children/m^2^ suggested that lower numbers of children/m^2^ was associated with increased step counts in boys and girls with more accumulated steps in girls [66]. Two further studies also assessed the average number of children/m^2^ and suggested that higher outdoor playground density non-significantly increased minutes spent in MVPA and decreased LPA [55]. Lahuerta-Contell et al. reported that more children/m^2^ was associated with more minutes/hour spent in VPA (β = 0.5 min/h, *p* = 0.01) [51]. Another study indicated that an outdoor area of 42.9m^2^/child was not associated with children’s MVPA levels [55]. Effect directions were not reported.

##### Sedentary time

**Fig. 6:**
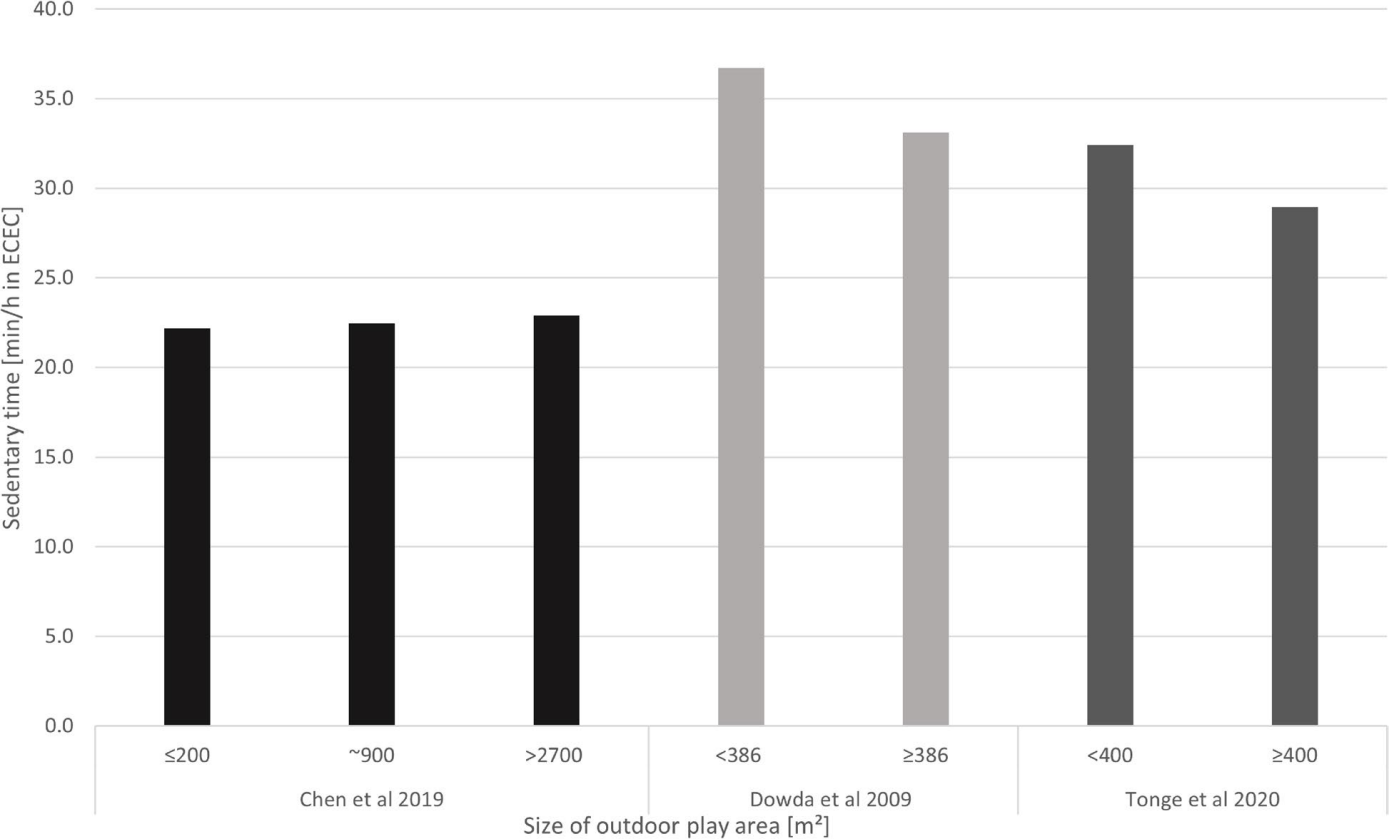
Sedentary time by size of outdoor play area

Based on a single cross-sectional study, the number of available outdoor play areas was not associated with a decrease in time spent sedentary [55], whereas outdoor play areas meeting the developmental needs of children aged 3 years and over was associated with reduced ST by 3.1 min/h (95% CI − 5.1 to − 1.2) [58]. The summary effect direction in Table 4 indicates that bigger outdoor play areas were associated with reduced ST. Figure 6 shows the minutes per hour sedentary for different outdoor play area sizes for studies where suitable data were available. The mean difference in ST between <386m^2^ and ≥ 386 m^2^ was − 3.6 min/h (95% CI -10.0 to 2.8) [26], between <400m^2^ and ≥ 400 m^2^ was − 3.5 min/h (95% CI − 6.2 to − 0.8) [25], and between ≤200m^2^ and > 2700 m^2^ was 0.71 min/h (95% CI − 0.9 to 2.3) [64]. Reducing the number of ECEC classes sharing the playground during recess time, which led to an increase in space per child from 7.4m^2^ to 16.7m^2^, was associated with a 1 min reduction (− 5.1% of recess time) in time spent sedentary [65]. More children/m^2^ was non-significantly associated with less time spent in sedentary in toddlers [67] and more sedentary time in preschoolers [51].

#### Use of outdoor space external to ECEC premises

##### Physical activity

Only one study investigated the association between using outdoor space external to ECEC premises and PA levels [69]. Researchers indicated an increased likelihood of more 6-year-old children’s TPA being in the 75th percentile when using a nearby park (adjusted odd ratio 1.45, 95% CI 1.16 to1.82). While no statistics for the adjusted model were reported for the 4- and 5-year-olds, the effect direction of the unadjusted model suggested a beneficial association between the proportion of children with PA levels above the 75th percentile and park use for 4-year-olds (OR = 1.32, 95% CI 0.53 to3.28) and a negative association for 5-year-olds (OR = 0.98, 95% CI 0.39 to2.41).

##### Sedentary time

Two studies assessed the association between using ECEC-external outdoor space and ST [68, 69]. The summary effect direction indicates that use of external outdoor space was associated with reduced ST. Unadjusted models suggested reduced time spent sedentary when using a nearby park for 4-year-olds (OR = 0.11, 95% CI 0.04 to0.30), 5-year-olds (OR = 0.64, 95% CI 0.26- to 1.57), and 6-year-olds (OR = 0.28 95% CI 0.11 to 0.73). Adjusted effect sizes were reported for 4-year-olds only, which also indicated reduced ST when using parks adjacent to ECECs (OR = 0.08, 95% CI 0.00 to 0.80) [69]. The frequency of nature visits was also associated with reduced ST (β = − 1.03, 95% CI − 1.80 to − 0.25) in Määttä et al., whereas the frequency of using playparks beyond ECEC premises was not associated with reduced ST (β = 0.26, 95%CI − 0.28 to 0.81) [68].

#### Outdoor play equipment

##### Physical activity

None of the included studies compared PA levels between availability/use of *portable* outdoor versus indoor play equipment. However, six studies assessed the association between portable outdoor play equipment and children’s MVPA levels. The effect direction synthesis suggested that portable outdoor play equipment at ECECs was associated with increased MVPA (Table 5). Gubbels et al. suggested that with each additional type of portable outdoor play equipment MVPA increased by 0.17% accelerometer wear time at ECECs [55]. When comparing ECECs with one or more portable outdoor play equipment and settings without any portable outdoor equipment, researchers found higher mean MVPA in settings with portable outdoor play equipment (7.4 min/d (SE 0.3) vs 6.2 min/d (SE 0.4)) [26, 27]. No summary statistics were reported in Copeland et al. but the authors indicated that the number of portable outdoor play equipment items was not significantly associated with time spent in MVPA [17]. Olesen et al. reported a non-significant negative correlation between the number of portable outdoor play equipment and % MVPA [56]. Another study investigated nine types of portable outdoor play items and indicted mixed findings in item’s ability to increase children’s MVPA and TPA. Only the presence of balls, portable slides and floor play equipment was associated with increased MVPA by 7.8 min/h, 8.4 min/h and 8.2 min/h ECEC time, respectively [27]. An increase in TPA by 13.4 min/h ECE time was found in relation to the presence of balls only. When assessing the total amount of portable equipment in the outdoor playground a non-significant association with increased TPA by 0.17 min/h (95% CI -0.22 to 0.56) was found by a further study [68]. Hannon and Brown assessed the change in LPA, MVPA and VPA as a function of percentage outdoor time before and after introducing portable outdoor play equipment to the ECEC over a duration of 5 days [70]. Researchers reported an increase in LPA by 3.5% (from 30.6 to 34.1%), MVPA by 7.8%, (from 9.8 to 17.6%) and VPA by 4.7% (from 2.3 to 7.0%). Boys showed a bigger before-after increase in time spent in LPA and MVPA than girls. However, the before-after difference for VPA was bigger in girls than boys [70].

**Table 5:** Comparison of portable outdoor play equipment at ECEC on MVPA and sedentary time

Study ID	Study Design	Sample size	Risk of bias	MVPA	Sedentary time
Copeland 2016 [17]	Cross-sectional	388	High	■	–
Dowda 2009^a^ [26]	Cross-sectional	299	Low	▲	▲
Gubbels 2018 [55]	Cross-sectional	281	Low	▲	■
Hannon & Brown 2008	Before-after	64	High	▲	▲
Ng 2020 [27]	Case-control	297	High	**◄►**	**–**
Olesen 2013 [56]	Cross-sectional	426	Low	▼	–
**Summary effect direction**				▲	▲

*Abbreviations*: *MVPA* Moderate-to-vigorous physical activity. ^a^ = Physical activity promoting v.s. not physical activity promoting ECEC (i.e. ≥ 1 piece of portable outdoor play equipment v.s. no presence of portable outdoor play equipment)

Effect direction: Study level: ▲ = portable outdoor play equipment benefits outcomes (lower sedentary time, higher physical activity); ▼ = portable outdoor play equipment harms outcomes (higher sedentary time, lower physical activity) ■ = (summary) statistics not presented

Summary: ▲ = studies show a beneficial association with portable outdoor play equipment at ECEC

Table 6 summarises the effect directions of the association between *fixed outdoor play equipment* and children’s physical activity. Across studies, the findings are inconclusive. Two studies indicated that the amount of fixed outdoor play equipment was associated with increased MVPA [56, 61]. Sugiyama et al. indicated that each additional piece of fixed outdoor play equipment was associated with an increase in MVPA by 2.2 min/ECE day (95% CI 0.4–3.9) [61]. In contrast, Dowda et al. suggested that having ≤8 pieces of fixed outdoor play equipment versus > 8 pieces of fixed outdoor play equipment was associated with higher mean MVPA of 7.6 min/h (SE 0.3) versus 6.4 min/g (SE 0.4) [26]. Another two studies did not report effect sizes but indicated that having less than 9 pieces of fixed outdoor play equipment was not significantly associated with time spent in MVPA [17], neither was the number of types of fixed outdoor equipment [55]. Assessing the presence of eight types of fixed outdoor play equipment, Ng et al. indicted that fixed tunnels reduced MVPA by 12.1 min/h whereas fixed sandboxes increased MVPA by 17.9 min/h. Effect directions for the remaining non-significant six items were not reported [27]. Similar findings were reported for TPA; presence of fixed tunnels reduced TPA by 12.9 min/day and availability of fixed sandboxes increased TPA by 19.8 min/day. Relating the total amount of fixed outdoor play equipment available at ECECs to TPA, another study found a non-significant association with lower TPA by 0.35 min/h [68].

**Table 6:** Comparison of fixed outdoor play equipment at ECEC on physical activity and sedentary time

Study ID	Study Design	Sample size	Risk of bias	Sedentary time	MVPA	TPA
Copeland 2016 [17]	Cross-sectional	388	High	–	■	–
Dowda 2009^a^ [26]	Cross-sectional	299	Low	▼	▼	–
Gubbels 2018 [55]	Cross-sectional	281	Low	–	■	–
Määttä 2019 [68]	Cross-sectional	778	Low	–	–	▼
Ng 2020 [27]	Case-control	297	High	–	**◄►**	**◄►**
Olesen 2013 [56]	Cross-sectional	426	Low	–	▲	–
Sugiyama 2012 [61]	Cross-sectional	107	High	▲	▲	–
**Summary effect direction**				**◄►**	**◄►**	**◄►**

*Abbreviations*: *MVPA* Moderate-to-vigorous physical activity, *TPA* Total physical activity. ^a^ = Physical activity promoting v.s. not physical activity promoting ECEC (i.e. ≤ 8 pieces of fixed outdoor play equipment v.s. > 8 pieces of fixed outdoor play equipment)

Effect direction: Study level: ▲ = fixed outdoor play equipment benefits outcomes (lower sedentary time, higher physical activity); ▼ = fixed outdoor play equipment harms outcomes (higher sedentary time, lower physical activity) ◄► = conflicting findings; ■ = (summary) statistics not presented; ‘-‘= outcome not assessed

Summary: ◄► conflicting or inconclusive findings

##### Sedentary time

Three studies assessed the association between *portable outdoor play equipment* and ST with an overall effect direction in favour of reduced ST (Table 5) [55, 70]. Dowda et al. indicated that having one or more portable outdoor play equipment versus no portable outdoor play equipment at ECECs resulted in lower mean ST of 33.4 (SE 0.8) min/h versus 36.7 (SE 1.5) min/h [26]. Hannon and Brown’s before-after assessment of adding portable outdoor play equipment suggested a decrease of ST by 16% of the total outdoor time (from 57.17 to 41.18%) with boys demonstrating a larger decrease of ST than girls [70]. While no summary statistics were reported in Gubbels et al. for time spent sedentary, authors reported that there was no significant association between number of portable outdoor play equipment and time spent sedentary during ECEC time [55].

Two studies were available with conflicting findings on the association between *fixed outdoor play equipment* and ST (Table 6) [26, 61]. While one study concluded that with each item of fixed outdoor play equipment ST reduces (β = − 4.4; 95% CI − 7.8 to − 1.1) [61], the other study indicated that having eight or fewer fixed outdoor play items reduces ST more than having more than eight items (− 32.3 min/h (SE 0.8) versus 35.8 min/h (SE 0.9) [26].

## Discussion

### Main findings

The objective of this study was to systematically review and synthesise the published literature on the association between childcare environment and practice and children’s PA levels, ST and injuries while attending centre based childcare settings. Findings suggested that while children spent more time in MVPA and LPA and less time sedentary when being outdoors compared to indoors, PA levels remained low and sedentary time high relative to time spent outdoors at ECECs. Studies from North America reported that girls were less active outdoors than boys, but studies from Scandinavia either didn’t find a gender difference or suggested that girls were more physically active. Findings were similar for studies that explored the association between engaging in outdoor versus indoor play and children’s PA levels and ST. These findings are consistent with Truelove et al. who conducted a meta-analysis on time spent physically active during outdoor play sessions. Researchers suggested that children spent an average of 10.7 min/h in MVPA, 25.5 min/h in TPA and 27.7 min/h sedentary [18]. The researchers concluded that children do accumulate more than a quarter of the recommended 180 minutes of daily physical activity during outdoor play sessions in ECECs [18].

Investigating the environmental characteristics of ECEC settings and its links to PA levels and ST revealed that having a dedicated outdoor play space is associated with more time spent in MVPA, although the benefit was minimal at only 1 min/h and 0.3% monitored time more compared to ECECs without dedicated outdoor play space. This finding is consistent with existing research. Lee et al.*’*s systematic review of over 85 studies identified, out of 287 potential correlates, that the number of play areas was positively associated with outdoor play, which included outdoor physical activity [71]. In addition to the availability of play areas, the size of the outdoor play area was shown to be of importance. Our data synthesis indicated that bigger outdoor play areas were associated with higher levels of MVPA and step counts compared to smaller outdoor play areas. A dose-response relationship with increased MVPA was observed with outdoor play areas up to ≥505m^2^ (5436 ft^2^). However, there was a threshold at outdoor play areas from 900m^2^ (9688 ft^2^) where no extra MVPA was accumulated. Considering the size of the children, larger outdoor play areas might not encourage more time in MVPA. Similar findings were observed for ST. However, the number of studies assessing the association between the size of the outdoor play area and time spent sedentary was too low to detect a dose-response relationship. Some ECECs find themselves in a position where there is no outdoor play area available at their premises. Using nearby outdoor play spaces such as parks might present a valuable solution. To date, only two studies investigated the use of ECEC-external outdoor play areas on children’s PA levels and ST. Studies indicated an association between using ECEC-external nature space and MVPA and ST. Based on the available evidence, increase in number of portable outdoor play equipment was associated with increased MVPA and reduced ST. Findings for fixed outdoor play equipment for PA and ST were inconclusive. The complexity of the child-environment relationship and ability to attribute characteristics of the outdoor environment of ECEC settings to child PA and sedentary behaviour has been noted in previous research [72, 73]. Outdoor play in open space environments were found to be associated with increased physical activity [73, 74] as well as greater availability of a wider variety of portable play equipment, and presence of certain fixed playground equipment [75].

Finally, none of the reviewed studies compared the rates or types of injury during outdoor PA to those during indoor PA in ECEC settings. On one hand this is somewhat surprising. Education settings routinely collect accident-related data, suggesting researchers would have had the opportunity to observe incidents or the recording of these. Conversely, injury-related factors were not identified as a correlate of outdoor PA and play among children aged 3 to 12 years in a recent systematic review [71].

Nevertheless, our finding is important. Previous research explored the injury incidence rates in 2105 ECECs in Norway, concluding that injuries were rare but more commonly occurring outdoors. Injuries were typically minor and more prevalent among boys than girls, with falls being the most common cause [28]. Current research in relation to injuries has primarily focused on outdoor *risky play*. Despite systematic review evidence indicating that *risky play* is not associated with injury risk in children aged 3 to 13 years [76] other research expounds the continued perceptions of parents, educators and decision-makers (as well as some researchers) that outdoor PA and play are inherently risky behaviours [30, 31, 77–79], which may impede their greater promotion and engagement. However, effective strategies can be imposed to mitigate risk [30, 33, 34], but should not preclude opportunities for activity. Furthermore, children are able to develop positive dispositions toward risk in appropriate environments if afforded the opportunity [80].

The erosion of outdoor and *risky play* in natural settings [31] emphasises the need to provide opportunities for children to engage in outdoor PA in the more managed environment of ECEC settings, while considering safety [77]. The onus is on the research community, however, to evidence the relative injury risk of outdoor versus indoor PA, so as to corroborate or breakdown risk-related arguments for and against outdoor PA and play.

### Certainty of the evidence

The quality of evidence across all exposure-outcome associations is reduced due to methodological bias and imprecision and because the majority of included studies were of cross-sectional design limiting the certainty in the observed associations. More than half of the studies across all exposures-outcome associations (except for use of ECEC-external outdoor space) where of high risk of bias. Limitations in the precision of the effect estimates were evident due to small sample sizes and a large number of ECEC settings were used to recruit the children. Large cluster sizes can impact the precision of effect estimates due to the similarity of children within one cluster and so the effective sample size being lower with increasing numbers of ECEC settings [81]. No evidence of selective reporting was detected, and publication bias could not be formally established due to less than 10 studies assessing the same exposure-outcome relationship using the same effect measures. There was also no evidence of unexplained heterogeneity for the assessed exposure-outcome associations. Heterogeneity could be explained by variability in and quality of exposure and outcome assessment. For example, assessment of outdoor/indoor time and play was primarily done using researcher-reported direct observations of children’s location. While most studies did not specify the tool used, the most common tools mentioned were the EPAO tool and the Observational System for Recording Activity in Preschoolers (OSRA-P) tool. Only one study relied on video recordings to capture where children spent their time [48] and another study used a device-based assessment for outdoor/indoor time (a Global Positioning System (GPS) device) [14]. The accuracy of estimating children’s location might be higher by using video recordings or location positioning systems compared to researcher or educator reported observation tool. However, the non-technological direct observation tools allow capturing children’s behaviours, social groupings, type of physical activities, and learning and environmental contexts. Therefore, they offer additional valuable information that could explain associations and so should be used in conjunction with video recordings or GPS devices. As for outcome assessment, heterogeneity was detected in terms of the models of accelerometers used, minimum valid wear time (1 day, 3 days, 7 days), definition of non-wear time and use of cut-offs to define PA intensities and ST.

### Strengths and limitations of the review process

This review built on previous evidence syntheses of correlates of outdoor play [71] and physical activity at ECEC [25], physical activity and sedentary time during outdoor play at ECEC [18] and outdoor time in general [82], and nature-based ECEC on physical, social, emotional and cognitive outcomes [83, 84]. This study advanced the current body of knowledge on PA and ST in childcare in that the location (outdoor vs indoor) and physical environmental characteristics and ECEC practices enabling outdoor and indoor PA were investigated in relation to accumulation of PA and ST.

This study followed the principles of PRISMA and Cochrane Collaboration’s recommendations for conducting evidence syntheses where a meta-analysis is not possible, avoiding a simple narrative description of individual study findings. Three relevant electronic databases were searched using a well-defined search string. To counterbalance the restricted number of databases searched, citation tracking was conducted. However, the completeness of the synthesised evidence might be impaired as the literature search was limited to peer-reviewed publications in English language for reasons of limited resources within the research team. This might have resulted in missing eligible studies in particular from low- and middle-income countries which might be published in other languages and databases. Although proportionate independent duplicate screening was employed for study selection to reduce study selection bias, not all studies were screened in duplicate which might have resulted in eligible studies being excluded.

### Implications for research, practice, and policy

Findings of this review highlight the importance of ECEC policies and practices to promote not only outdoor time but also engagement in activities and availability of resources such as portable play equipment that enable children to be physically active for a sustained amount of time while outdoors. Childcare settings with limited availability of suitable outdoor play areas at their premises should be encouraged to consider using nearby outdoor play areas with or without natural space. The size of outdoor play areas appears to play a role for children’s ability to be physically active over a longer period of time, but further research is needed to establish the optimal size of the play area. Identifying the dose-response relationship by treating the size of the outdoor space as continuous variable rather than using threshold categories is important as it has the potential to influence the planning and building of future and restructuring of existing childcare settings. Using continuous measures of the outdoor play area would require involving a larger number of ECEC settings than the included studies of this review did which calls for a large-scale country-wide assessment of ECEC outdoor spaces. Future research should consider using and reporting a suitable direct observation tool in combination with technological devices (e.g., GPS) to reliably assess children’s location outdoors and indoors in the ECEC setting. Efforts should be made to identify the underlying reasons for girls’ lower PA levels outdoors and to develop strategies that would encourage girls to be more active when spending time outdoors at ECEC. Furthermore, research is urgently required to ascertain the relative injury risk profile of outdoor versus indoor PA and play.

ECEC educators should be educated about the relative risks of outdoor and indoor PA, and in turn be supported in educating parents about this. Decision-makers should support ECEC settings when providing outdoor PA opportunities that are conducted in the best interests of children’s development. The benefits of outdoor time and play opportunities at ECEC; however, go beyond increasing TPA and MVPA and decreasing ST. Improvements in cognitive and behavioural outcomes [83–86] and immunoregulation have been observed [87] which could be achieved independently of PA levels. Therefore, one might argue that outdoor play time at ECEC settings serves multiple important purposes for child development and that its contribution for children meeting the physical activity guidelines is limited. Opportunities for healthy child development are influenced by a complex array of factors. Our findings suggest that programmes to alter environmental and practice-related factors of ECEC settings and provision are only likely to be a small part of the solution to increase PA and decrease ST in young children. Alternatively, a systems-focused approach that identifies the leverage points at which small alterations can be amplified to produce large and sustainable changes in behaviours is likely to be required. The available evidence suggests factors such as outdoor provision, space or portable equipment create only small differences.

## Conclusion

This systematic review identified several physical environmental characteristics and ECEC practices that contribute to accumulation of children’s PA and ST indoor and outdoor. ECEC policies and practices should promote not only outdoor time but also engagement in active play and availability of resources such as portable play equipment and sufficient size of outdoor play areas that enable children to be physically active for a sustained amount of time while outdoors. Evidence is lacking and thus research urgently required to ascertain the relative injury risk profile of outdoor versus indoor PA to corroborate or breakdown risk-related arguments for and against outdoor PA and play by educators and parents.

## Supplementary Information

**Additional file 1.** Deviations from the registered protocol. – This document provides justifications for changes made to the originally registered review protocol.

**Additional file 2.** Electronic database search strings. – This document details the search string run in Medline, PsycINFO and SPORTDiscuss.

**Additional file 3.** CASP modification and results. – This document details modifications made to the CASP risk of bias tools, and the results of assessments for individual studies.

**Additional file 4.** Study characteristics tables and accelerometer cut points. – This document summarises the characteristics of included studies by exposure type. It also details accelerometer cut points used in the included studies.

**Additional file 5.** PRISMA 2020 Checklist.

## Data Availability

All information is included in the manuscript and accompanying files.

## Abbreviations

CCS: Case control studies
CI: Confidence interval
ECEC: Early childhood education and care
LMVPA: Light-to-vigorous intensity physical activity
LPA: Light intensity physical activity
min/h: Minutes per hour
MVPA: Moderate-to-vigorous intensity physical activity
m^2^: Square meter
TPA: Total physical activity
OR: Odd ratio
PA: Physical activity
SD: Standard deviation
ST: Sedentary time
VPA: Vigorous intensity physical activity
