# Supplementary material for: Environmental and practice factors associated with children’s device-measured physical activity and sedentary time in early childhood education and care centres: a systematic review
Source: Int J Behav Nutr Phys Act. 2022 Jul 14;19:84. doi: 10.1186/s12966-022-01303-2 (PMC9284804; doi:10.1186/s12966-022-01303-2)
Supplement: Supplementary file 1 — Additional file 1. Deviations from the registered protocol. – This document provides justifications for changes made to the originally registered review protocol. [file 12966_2022_1303_MOESM1_ESM.docx]

**Additional file 1.** Deviations from the registered protocol

PROSPERO registration number: CRD42020189886 (15 Jun 20)

| **Original protocol submission** | **Deviation as reported in manuscript** | **Justification** | **Page** |
| --- | --- | --- | --- |
| **Review question:**  Primary research questions:  1. How much PA do children obtain outdoors compared to indoors while attending early childhood education and care settings?  2. How do patterns differ by PA intensities between time spent indoors and outdoors while attending early childhood education and care settings?  Secondary research questions:  3. How much time do children spent being sedentary outdoors compared to indoors while attending early childhood education and care settings?  4. In studies measuring PA, are there more or different types of injury in young children during outdoor PA compared to indoor PA while attending early childhood education and care settings? | The aim of this study was threefold: to systematically review and synthesise the published evidence (1) investigating how much PA children obtain, and how much time they spend sedentary, outdoors compared to indoors while attending ECEC, and how PA patterns differ by PA intensity and ST; (2) assessing the influence of the physical environment and practices on children's PA and ST, and (3) addressing if are there more or different types of injury in young children during outdoor PA compared to indoor PA while attending ECEC. | In the development of this review, it was determined that a sole focus on comparing indoor versus outdoor PA provided insufficient novelty to the evidence-base. The additional synthesis of the relationship between environmental factors and PA/ST facilitated the development of more meaningful policy and practice recommendations. | 7 |
| **Searches:**  We will search … for subject headings and keywords relating to … exposure and comparator (*i.e.* outdoor and indoor PA) … | **Search strategy and selection process:**  We will search … for subject headings and keywords relating to … exposure and comparator (*i.e.* use of outdoor and indoor space) … | The proposed search strategy was updated with additional subject headings and keywords relating to exposures and comparators that reflected the altered aims of the final review. | 9 |
| **Types of study to be included:**  Cohort studies (cross-sectional and longitudinal) regardless whether outcomes have been assessed in the same child indoors and outdoors, or case-control (i.e. non-randomised controlled before & after study) or (cluster) randomised controlled trials comparing the effect of indoor PA with outdoor PA. | **Eligibility criteria (study design):**  Cohort studies (cross-sectional and longitudinal) irrespective of whether outcomes were assessed in the same child both indoors and outdoors, case-control studies (*i.e.* non-randomised controlled before and after studies), or (cluster) randomised controlled trials (RCTs). | Given the broader aims of the final review, the inclusion criterion for study design was amended so as not to restrict the literature search to (cluster) RCTs that only compared the effect of indoor PA with outdoor PA, allowing for additional ECEC environment-related papers to be examined. | 8 |
| **Intervention(s), exposure(s):**  Structured and unstructured (free play) PA in any outdoor space. | **Eligibility criteria (exposure):**  Use of outdoor space including, but not limited to, the factors of time, availability, play, size and fixed and portable outdoor equipment. | The exposure-related eligibility criterion was expanded to reflect the altered aims of the research review. | 8 |
| **Comparator(s)/control:**  Indoor structured and unstructured (free play) PA. | **Eligibility criteria (comparator):**  Where applicable, the use of indoor space including, but not limited to, the factors of time and play. | The comparator-related eligibility criterion was expanded to reflect the altered aims of the research review. | 8 |
| **Strategy for data synthesis:**  A sensitivity analysis will be conducted where low quality studies will be removed from the synthesis to establish if they influenced the overall result for a certain outcome. | N/A | Sensitivity analysis was not performed due to insufficient data. | N/A |
