# Supplementary material for: Environmental and practice factors associated with children’s device-measured physical activity and sedentary time in early childhood education and care centres: a systematic review
Source: Int J Behav Nutr Phys Act. 2022 Jul 14;19:84. doi: 10.1186/s12966-022-01303-2 (PMC9284804; doi:10.1186/s12966-022-01303-2)
Supplement: Supplementary file 2 — Additional file 2. Electronic database search strings. – This document details the search string run in Medline, PsycINFO and SPORTDiscuss. [file 12966_2022_1303_MOESM2_ESM.docx]

**Additional file 2. Search strings for Ovid Medline, PsychInfo (EBSCOhost), SPORTDiscus (EBSCOhost)**

Ovid MEDLINE(R) and Epub Ahead of Print, In-Process & Other Non-Indexed Citations and Daily 1946 to June 02, 2020

- Searched on 04.06.2020
- Resulted in 434 records

Ovid MEDLINE(R) and Epub Ahead of Print, In-Process, In-Data-Review & Other Non-Indexed Citations, Daily and Versions 1946 to March 24, 2022

- Searched 25.03.2022
- Resulted in 125 records

1. preschool child/

2. (preschooler* or pre-schooler*).tw.

3. "pre schooler".tw.

4. preschool-aged.tw.

5. toddler*.tw.

6. (young adj child*).tw.

7. "Early childhood".tw.

8. "early years".tw.

9. or/1-8

10. exp child care/

11. child day care centers/

12. Schools, Nursery/

13. (kindergarten* or nurser* or preschool*).tw.

14. (kinder-garten* or pre-school* or preprimary or pre-primary).tw.

15. "early learning centre".tw.

16. "early learning center".tw.

17. "centre-based childcare".tw.

18. "center-based care".tw.

19. "childcare centre".tw.

20. "childcare center".tw.

21. "early care and education".tw.

22. or/10-21

23. exp Exercise/

24. exp "Play and Playthings"/

25. "active play".tw.

26. (physical* adj activ*).tw.

27. (movement or exercise).tw.

28. ((locomotor or motor) adj2 activ*).tw.

29. ((indoor or outdoor) adj activ*).tw.

30. "outdoor play".tw.

31. ((free or unstructured or structured) adj play).tw.

32. "physical play".tw.

33. "outdoor time".tw.

34. "indoor time".tw.

35. 23 or 24 or 25 or 26 or 27 or 28 or 29 or 30 or 31 or 32 or 33 or 34

36. exp Accelerometry/

37. (accelerometer* or actigraph or activPAL).tw.

38. ((motor or activity) adj (sensor or device or measurement)).tw.

39. "activity monitor".tw.

40. "activity tracker".tw.

41. pedometer*.tw.

42. "CSA monitor".tw.

43. 36 or 37 or 38 or 39 or 40 or 41 or 42

44. 9 and 22 and 35

45. 9 and 22 and 35 and 43

46. limit 45 to (english language and yr="1997 -Current" and journal article)

47. limit 45 to (english language and yr="2020 -Current" and journal article)

**PsycInfo search 25.03.2022**

| S37 | S9 AND S27 AND S28 limited to 2020 onwards and academic journals | (53) |
| --- | --- | --- |
| S36 | S9 AND S27 AND S28 | (467) |
| S35 | S29 OR S30 OR S31 OR S32 OR S33 OR S34 | (8,686) |
| S34 | TI CSA monitor OR AB CSA monitor | (8) |
| S33 | TI pedometer OR AB pedometer | (899) |
| S32 | TI ( activity tracker or fitness trackers or wearable technology ) OR AB ( activity tracker or fitness trackers or wearable technology ) | (617) |
| S31 | TI activity monitor OR AB activity monitor | (1,488) |
| S30 | TI (motor or activity) N2 (sensor or device or measurement) OR AB (motor or activity) N2 (sensor or device or measurement) | (2,073) |
| S29 | TI ( accelerometer* or actigraph or activPAL ) OR AB ( accelerometer* or actigraph or activPAL ) | (4,171) |
| S28 | S19 OR S20 OR S21 OR S22 OR S23 OR S24 OR S25 OR S26 | (81,342) |
| S27 | S10 OR S11 OR S12 OR S13 OR S14 OR S15 OR S16 OR S17 OR S18 | (36,965) |
| S26 | TI indoor N1 time OR AB indoor N1 time | (78) |
| S25 | TI outdoor play OR AB outdoor play | (472) |
| S24 | TI locomotor N1 activity OR AB locomotor N1 activity | (11,050) |
| S23 | TI physical N1 activity OR AB physical N1 activity | (39,534) |
| S22 | DE "Physical Activity" | (23,398) |
| S21 | TI "active play" OR AB "active play" | (241) |
| S20 | DE "Toys" OR DE "Childhood Play Behavior" | (8,590) |
| S19 | DE "Exercise" | (33,684) |
| S18 | TI early care N1 education OR AB early care N1 education | (1,478) |
| S17 | TI ( "childcare centre" or "childcare center" ) OR AB ( "childcare centre" or "childcare center" ) | (129) |
| S16 | TI ( "centre-based childcare" or "center-based childcare". ) OR AB ( "centre-based childcare" or "center-based childcare". ) | (70) |
| S15 | TI ( "early learning center or "early learning centre" ) OR AB ( "early learning center or "early learning centre" ) | (1) |
| S14 | TI ( kindergarten* or nurser* or preschool*or preprimary or pre-primary) OR AB ( kindergarten* or nurser* or preschool*or preprimary or pre-primary ) | (23,432) |
| S13 | DE "Kindergarten Students" OR DE "Kindergartens" | (8,988) |
| S12 | DE "Nursery School Students" OR DE "Nursery Schools" | (945) |
| S11 | DE "Child Day Care" | (2,493) |
| S10 | DE "Child Care" | (8,899) |
| S9 | S1 OR S2 OR S3 OR S4 OR S5 OR S6 OR S7 OR S8 | (61,106) |
| S8 | TI "early years". OR AB "early years". | (4,775) |
| S7 | TI "early years". OR AB "early years". | (4,775) |
| S6 | TI "early childhood" OR AB "early childhood" | (27,874) |
| S5 | AB (young N child*) OR TI (young N child*) | (509) |
| S4 | TI toddler* OR AB toddler* | (10,175) |
| S3 | TI preschool-aged OR AB preschool-aged | (2,494) |
| S2 | TI ( (preschooler* or pre-schooler*) ) OR AB ( (preschooler* or pre-schooler*) ) | (13,959) |
| S1 | DE "Preschool Students" | (12,235) |

**PsycInfo Search 5^th^ June 2020.**

| **#** | **Query** | **Results** |
| --- | --- | --- |
| S38 | S9 AND S27 AND S28 limited to 1997onwards | 7 |
| S37 | S9 AND S27 AND S28 | 394 |
| S36 | S9 AND S27 AND S28 AND S35 | 28 |
| **S35** | **S29 OR S30 OR S31 OR S32 OR S33 OR S34** | **7,380** |
| S34 | TI CSA monitor OR AB CSA monitor | 7 |
| S33 | TI pedometer OR AB pedometer | 833 |
| S32 | TI ( activity tracker or fitness trackers or wearable technology ) OR AB ( activity tracker or fitness trackers or wearable technology ) | 372 |
| S31 | TI activity monitor OR AB activity monitor | 1,317 |
| S30 | TI (motor or activity) N2 (sensor or device or measurement) OR AB (motor or activity) N2 (sensor or device or measurement) | 1,878 |
| S29 | TI ( accelerometer* or actigraph or activPAL ) OR AB ( accelerometer* or actigraph or activPAL ) | 3,456 |
| **S28** | **S19 OR S20 OR S21 OR S22 OR S23 OR S24 OR S25 OR S26** | **72,112** |
| **S27** | **S10 OR S11 OR S12 OR S13 OR S14 OR S15 OR S16 OR S17 OR S18** | **34,366** |
| S26 | TI indoor N1 time OR AB indoor N1 time | 61 |
| S25 | TI outdoor play OR AB outdoor play | 397 |
| S24 | TI locomotor N1 activity OR AB locomotor N1 activity | 10,529 |
| S23 | TI physical N1 activity OR AB physical N1 activity | 34,223 |
| S22 | DE "Physical Activity" | 19,682 |
| S21 | TI "active play" OR AB "active play" | 213 |
| S20 | DE "Toys" OR DE "Childhood Play Behavior" | 7,761 |
| S19 | DE "Exercise" | 29,272 |
| S18 | TI early care N1 education OR AB early care N1 education | 1,175 |
| S17 | TI ( "childcare centre" or "childcare center" ) OR AB ( "childcare centre" or "childcare center" ) | 110 |
| S16 | TI ( "centre-based childcare" or "center-based childcare". ) OR AB ( "centre-based childcare" or "center-based childcare". ) | 59 |
| S15 | TI ( "early learning center or "early learning centre" ) OR AB ( "early learning center or "early learning centre" ) | 10 |
| S14 | TI ( kindergarten* or nurser* or preschool*or preprimary or pre-primary) OR AB ( kindergarten* or nurser* or preschool*or preprimary or pre-primary ) | 21,821 |
| S13 | DE "Kindergarten Students" OR DE "Kindergartens" | 8,210 |
| S12 | DE "Nursery School Students" OR DE "Nursery Schools" | 921 |
| S11 | DE "Child Day Care" | 2,418 |
| S10 | DE "Child Care" | 8,184 |
| **S9** | **S1 OR S2 OR S3 OR S4 OR S5 OR S6 OR S7 OR S8** | **55,042** |
| S8 | TI "early years". OR AB "early years". | 4,291 |
| S7 | TI "early years". OR AB "early years". | 4,291 |
| S6 | TI "early childhood" OR AB "early childhood" | 24,522 |
| S5 | AB (young N child*) OR TI (young N child*) | 427 |
| S4 | TI toddler* OR AB toddler* | 9,166 |
| S3 | TI preschool-aged OR AB preschool-aged | 2,187 |
| S2 | TI ( (preschooler* or pre-schooler*) ) OR AB ( (preschooler* or pre-schooler*) ) | 12,804 |
| S1 | DE "Preschool Students" | 11,099 |

**SPORTDiscuss 5^th^ June 2020**

S48 S44 AND S45 Limiters – Published Date: 19970101-20201231

Narrow by Language: english

S47 S44 AND S45 Narrow by Language: -english

S46 S44 AND S45

S45 S11 AND S20 AND S33

S44 S34 OR S35 OR S36 OR S37 OR S38 OR S39 OR S40 OR S41 OR S42 OR S43

S43 AB "device-based physical activity"

S42 TI "device-based physical activity"

S41 AB ("activity monitors" OR "activity sensors" OR "activity trackers" OR "activity measurements")

S40 AB ("activity monitor" OR "activity sensor" OR "activity tracker" OR "activity measurement")

S39 TI ("activity monitors" OR "activity sensors" OR "activity trackers" OR "activity measurements")

S38 TI ("activity monitor" OR "activity sensor" OR "activity tracker" OR "activity measurement")

S37 AB (accelerometer* OR actigraph* OR activpal OR pedometer*)

S36 TI (accelerometer* OR actigraph* OR activpal OR pedometer*)

S35 SU pedometer

S34 SU accelerometry Expanders - Apply

S33 S21 OR S22 OR S23 OR S24 OR S25 OR S26 OR S27 OR S28 OR S29 OR

S30 OR S31 OR S32

S32 AB ("outdoor play" OR "outdoor time" OR "indoor play" OR "indoor time")

S31 TI ("outdoor play" OR "outdoor time" OR "indoor play" OR "indoor time")

S30 AB ("physical activity" OR "physically active" OR "active play" OR "physically active play" OR "physical play")

S29 TI ("physical activity" OR "physically active" OR "active play" OR "physically active play" OR "physical play")

S28 AB exercis*

S27 TI exercis*

S26 SU indoor games Expanders - Apply

S25 SU outdoor education Expanders - Apply

S24 SU movement

S23 SU play

S22 SU exercise

S21 SU physical activity

S20 S12 OR S13 OR S14 OR S15 OR S16 OR S17 OR S18 OR S19

S19 AB "early childhood education"

S18 TI "early childhood education"

S17 AB ("early learning center" OR "early learning centre" OR "child daycare" OR "center based childcare" OR "centre based childcare" OR "center-based childcare" OR "centrebased childcare" OR

"childcare center" OR "childcare centre")

S16 TI ("early learning center" OR "early learning centre" OR "child daycare" OR "center based childcare" OR "centre based childcare" OR "center-based childcare" OR "centrebased childcare" OR "childcare center" OR "childcare centre")

S15 AB (kindergarten* OR nurser* OR preschool OR preschools OR

preprimary OR preschool OR pre-primary OR pre-schools)

S14 TI (kindergarten* OR nurser* OR preschool OR preschools OR preprimary OR preschool OR pre-primary OR pre-schools)

S13 SU childcare

S12 SU early childhood

S11 S1 OR S2 OR S3 OR S4 OR S5 OR S6 OR S7 OR S8 OR S9 OR S10

S10 SU toddler

S9 SU kindergarten children

S8 AB "early childhood"

S7 TI "early childhood"

S6 AB ("young children" OR "young child" OR "early years")

S5 TI ("young children" OR "early years")

S4 TI "young child"

S3 AB (preschooler* OR preschooler* OR toddler* OR preschool-aged)

S2 TI (preschooler* OR preschooler* OR toddler* OR preschool-aged)

S1 SU preschool children

**SPORTDiscuss 31^st^ March 2022**

S46 S11 AND S20 AND S33 AND S44 Limiters – Published Date: 20200101-20221231

S45 S11 AND S20 AND S33 AND S44

S44 S34 OR S35 OR S36 OR S37 OR S38 OR S39 OR S40 OR S41 OR S42 OR S43

S43 AB "device-based physical activity"

S42 TI "device-based physical activity"

S41 AB ("activity monitors" OR "activity sensors" OR "activity trackers" OR "activity measurements")

S40 AB ("activity monitor" OR "activity sensor" OR "activity tracker" OR "activity measurement")

S39 TI ("activity monitors" OR "activity sensors" OR "activity trackers" OR "activity measurements")

S38 TI ("activity monitor" OR "activity sensor" OR "activity tracker" OR "activity measurement")

S37 AB (accelerometer* OR actigraph* OR activpal OR pedometer*)

S36 TI (accelerometer* OR actigraph* OR activpal OR pedometer*)

S35 SU pedometer

S34 SU accelerometry Expanders - Apply

S33 S21 OR S22 OR S23 OR S24 OR S25 OR S26 OR S27 OR S28 OR S29 OR

S30 OR S31 OR S32

S32 AB ("outdoor play" OR "outdoor time" OR "indoor play" OR "indoor time")

S31 TI ("outdoor play" OR "outdoor time" OR "indoor play" OR "indoor time")

S30 AB ("physical activity" OR "physically active" OR "active play" OR "physically active play" OR "physical play")

S29 TI ("physical activity" OR "physically active" OR "active play" OR "physically active play" OR "physical play")

S28 AB exercis*

S27 TI exercis*

S26 SU indoor games Expanders - Apply

S25 SU outdoor education Expanders - Apply

S24 SU movement

S23 SU play

S22 SU exercise

S21 SU physical activity

S20 S12 OR S13 OR S14 OR S15 OR S16 OR S17 OR S18 OR S19

S19 AB "early childhood education"

S18 TI "early childhood education"

S17 AB ("early learning center" OR "early learning centre" OR "child daycare" OR "center based childcare" OR "centre based childcare" OR "center-based childcare" OR "centrebased childcare" OR

"childcare center" OR "childcare centre")

S16 TI ("early learning center" OR "early learning centre" OR "child daycare" OR "center based childcare" OR "centre based childcare" OR "center-based childcare" OR "centrebased childcare" OR "childcare center" OR "childcare centre")

S15 AB (kindergarten* OR nurser* OR preschool OR preschools OR

preprimary OR preschool OR pre-primary OR pre-schools)

S14 TI (kindergarten* OR nurser* OR preschool OR preschools OR preprimary OR preschool OR pre-primary OR pre-schools)

S13 SU childcare

S12 SU early childhood

S11 S1 OR S2 OR S3 OR S4 OR S5 OR S6 OR S7 OR S8 OR S9 OR S10

S10 SU toddler

S9 SU kindergarten children

S8 AB "early childhood"

S7 TI "early childhood"

S6 AB ("young children" OR "young child" OR "early years")

S5 TI ("young children" OR "early years")

S4 TI "young child"

S3 AB (preschooler* OR preschooler* OR toddler* OR preschool-aged)

S2 TI (preschooler* OR preschooler* OR toddler* OR preschool-aged)

S1 SU preschool children
