# Supplementary material for: Environmental and practice factors associated with children’s device-measured physical activity and sedentary time in early childhood education and care centres: a systematic review
Source: Int J Behav Nutr Phys Act. 2022 Jul 14;19:84. doi: 10.1186/s12966-022-01303-2 (PMC9284804; doi:10.1186/s12966-022-01303-2)
Supplement: Supplementary file 3 — Additional file 3. CASP modification and results. – This document details modifications made to the CASP risk of bias tools, and the results of assessments for individual studies. [file 12966_2022_1303_MOESM3_ESM.docx]

**Additional file 3.** Modification of the CASP tools and Risk of bias ratings of included studies

**Modifications of the CASP tool:**

For RCTs, sections A (validity of the basic design), B (methodological soundness) and selected items from C (results) of the tool were used. The Section C item regarding the benefits and costs of the experimental intervention was excluded. An additional item (accuracy of outcome measurement) was added to section B of both the RCT and case-control study tools. In applying the cohort study tool, items regarding follow-up were replaced with a single item concerning the completeness of data (*i.e.* accounting for attrition and missing data). For each tool, item 1 (clear study aim) and section C (or D for RCTs), which concerned the application of results, were excluded from the overall risk of bias assessment.

**Modified version of the CASP tool for randomised controlled trials:**

|  | **Section A: Is the basic study design valid for an RCT?** | | **Section B: Was the study methodologically sound?** | | | | **Section C: What are the results?** | | **Rating** | |
| --- | --- | --- | --- | --- | --- | --- | --- | --- | --- | --- |
| **Report authors and date** | **Was the assignment to groups randomised?** | **Were all of the participants who entered the trial accounted for at conclusion?** | **Were participants, ECEC staff and study personnel ‘blind’ to the intervention?** | **Were the groups similar at the start of the trial?** | **Aside from the experimental intervention, were the groups treated equally?** | **Was the outcome accurately measured to minimise bias?** | **Was the treatment effect large?** | **Was the estimate of the treatment effect precise?** | **Total number of low risk of bias items** | **Overall risk of bias assessment*** |
| Hannon and Brown 2008 | No | No | No | N/A | N/A | Can’t tell | Yes | Can’t tell | 1 | High |
| Saunders *et al.* 2019 | Yes | Yes | No | No | Yes | Can’t tell | No | Can’t tell | 3 | High |
| Trost *et al.* 2008 | Yes | Yes | Can’t tell | Yes | Yes | Can’t tell | Can’t tell | Yes | 5 | Low |
| Van Cauwenberghe *et al.* 2012 | No | Can’t tell | No | N/A | N/A | Can’t tell | Can’t tell | Can’t tell | 0 | High |

***** Studies with > 50% of ‘yes’ responses were deemed to be at low risk of bias

**Modified version of the CASP tool for case control studies:**

|  | **Section A: Are the results of the trial valid?** | | | | | | | **Section B: What are the results?** | | | **Rating** | |
| --- | --- | --- | --- | --- | --- | --- | --- | --- | --- | --- | --- | --- |
| **Report authors and date** | **Did the authors use an appropriate method to answer their question?** | **Were the cases recruited through random sampling methods?** | **Were the controls selected in an appropriate way?** | **Was the exposure accurately measured to minimise bias?** | **Was the outcome accurately measured to minimise bias?** | **Aside from the experimental intervention, were groups treated equally?** | **Have authors accounted for potential confounding factors in the design and/or analysis?** | **Was the treatment effect large?** | **Was the estimate of the treatment effect precise?** | **Are the results trustworthy?** | **Total number of low risk of bias items** | **Overall risk of bias assessment*** |
| Ng *et al.* 2020 | Yes | Yes | Can’t tell | Can’t tell | Can’t tell | Yes | Yes | Can’t tell | Can’t tell | Can’t tell | 4 | High |

* > 50% of ‘yes’ responses were deemed to be at low risk of bias

**Modified version of the CASP tool for cohort studies:**

|  | **Section A: Are the results of the study valid?** | | | | | | **Section B: What are the results?** | | **Rating** | |
| --- | --- | --- | --- | --- | --- | --- | --- | --- | --- | --- |
| **Report authors and date** | **Was the cohort recruited via random sampling methods?** | **Was the exposure accurately measured to minimise bias?** | **Was the outcome accurately measured to minimise bias?** | **Have the authors identified all important cofounding factors?** | **Have they taken account of confounding factors in the design and/or analysis?** | **Was the data complete enough (attrition and missing data)?** | **Are the results precise?** | **Are the results trustworthy?** | **Total number of low risk of bias items** | **Overall risk of bias assessment*** |
| Anderson *et al.* 2017 | Yes | No | Yes | No | Yes | Yes | Yes | No | 5 | Low |
| Barbosa *et al.* 2016 | Yes | Can’t tell | Yes | No | Yes | Yes | No | Yes | 5 | Low |
| Bell *et al.* 2015 | Yes | Can’t tell | Yes | No | Yes | Yes | Can’t tell | Yes | 5 | Low |
| Boldeman *et al.* 2011 | Can’t tell | Can’t tell | Yes | Yes | Yes | Yes | Can’t tell | Can’t tell | 4 | High |
| Cardon *et al.* 2008 | Yes | Can’t tell | Yes | No | Yes | Yes | Can’t tell | No | 4 | High |
| Chen *et al.* 2020 | Can’t tell | Can’t tell | Yes | No | Yes | Yes | Yes | Can’t tell | 4 | High |
| Copeland *et al.* 2016 | Yes | Can’t tell | No | Yes | Yes | Yes | Can’t tell | No | 4 | High |
| Dowda *et al.* 2009 | Can’t tell | Can’t tell | Yes | No | Yes | Yes | Yes | Yes | 5 | Low |
| Gubbels *et al.* 2018 | Yes | Yes | Can’t tell | Yes | Yes | Yes | Can’t tell | Can’t tell | 5 | Low |
| Henderson *et al.* 2015 | Yes | Yes | No | Yes | Yes | Yes | Yes | Yes | 7 | Low |
| Hinkley *et al.* 2016 | Yes | Can’t tell | Can’t tell | No | Yes | Yes | Can’t tell | Can’t tell | 3 | High |
| Lahuerta-Contell *et al.* 2021 | Can't Tell | Can't Tell | Yes | No | Yes | Yes | Can't Tell | Yes | 4 | High |
| Määttä *et al.*  2019 | Yes | Can’t tell | Yes | No | Yes | Can’t tell | Yes | Yes | 5 | Low |
| Mazzucca *et al.* 2018 | Can’t tell | Yes | Yes | Can’t tell | Yes | Can’t tell | Can’t tell | Can’t tell | 3 | High |
| Olesen *et al.* 2013 | Yes | Can’t tell | Yes | Yes | Yes | Yes | Yes | Yes | 7 | Low |
| Raustorp *et al.* 2012 | Can’t tell | Can’t tell | Yes | No | No | Yes | No | No | 2 | High |
| Schlechter *et al.* 2017 | Yes | Yes | Can’t tell | No | No | Yes | Yes | Can’t tell | 4 | High |
| Stephens *et al.* 2014 | Yes | Can’t tell | No | Yes | Yes | Yes | Yes | Yes | 6 | Low |
| Sugiyama *et al.* 2012 | Can’t tell | Can’t tell | Can’t tell | Yes | Yes | Yes | No | Can’t tell | 3 | High |
| Tandon *et al.* 2015 | Can’t tell | Yes | Can’t tell | No | Yes | Can’t tell | Can’t tell | Can’t tell | 2 | High |
| Tandon *et al.* 2018 | Can’t tell | Yes | Yes | No | Can’t tell | Can’t tell | Yes | No | 3 | High |
| Tonge *et al.* 2020 | Can’t tell | Can’t tell | Yes | No | Yes | Can’t tell | No | Can’t tell | 2 | High |
| Vanderloo *et al.* 2013 | Can’t tell | Yes | Yes | Yes | Yes | Can’t tell | Can’t tell | Yes | 5 | Low |
| Vega-Perona *et al.* 2022 | No | Can't Tell | Can't Tell | No | Yes | Yes | No | Can't Tell | 2 | High |
| Zhang *et al.* 2021 | Can't Tell | Yes | Yes | No | Yes | Yes | No | Can't Tell | 4 | High |

* Studies with > 50% of ‘yes’ responses were deemed to be at low risk of bias
