# Supplementary material for: Environmental and practice factors associated with children’s device-measured physical activity and sedentary time in early childhood education and care centres: a systematic review
Source: Int J Behav Nutr Phys Act. 2022 Jul 14;19:84. doi: 10.1186/s12966-022-01303-2 (PMC9284804; doi:10.1186/s12966-022-01303-2)
Supplement: Supplementary file 4 — Additional file 4. Study characteristics tables and accelerometer cut points. – This document summarises the characteristics of included studies by exposure type. It also details accelerometer cut points used in the included studies. [file 12966_2022_1303_MOESM4_ESM.docx]

**Additional File 4.** Study characteristics tables

**Table 1. Characteristics of studies comparing physical activity levels and sedentary time during time spent outdoors and indoors**

| **Author, year, country** | **Study design** | **Participants: n, age, gender** | **Exposure description** | **Comparison description** | **Outcome assessment** | **Outcomes**  **[unit]** | **Covariates** | **Statistical analysis** | **Percentage complete data** |
| --- | --- | --- | --- | --- | --- | --- | --- | --- | --- |
| Andersen *et al.* 2017  Norway | Cross-sectional | **Sample:**  n = 116  n preschools = 11  **Age:**  Range = 3-4 years  Mean = 3.7 ± 0.4 years  **Gender:**  57% girls | Staff logged child location and outdoor time rating for each hour on a four-point scale: 0-25%, 26-50%, 51-75%, 76-100%. | Percentage indoor time logged. | **Devices:** ActiGraph GT1M and GT3X+ accelerometers.  **Epoch:** 15s.  **Valid data:** 6h/day for at least 2 days.    **Non-wear time:** Periods with value of 0 for 60min.    **Cut-points:** Butte *et al*. 2014 | TPA  [cpm] | Wear time, gender, age, preschool. | Independent and paired t-tests.  Univariate general linear model for adjustment. | 95.7% |
| Copeland *et al.* 2016  USA | Cross- sectional | **Sample:**  n = 388  n childcare centres = 30  **Age:**  Range: 3-6 years  Mean: 4.3 ±0.7 years  **Gender:**  51% girls. | Times and durations spent outdoors were recorded by the researcher. | Times and durations spent indoors were recorded by the researcher. | **Device:** ActiCal uniaxial accelerometer  **Epoch:** 15s    **Valid data:** 24 hours excluding sleep and non-wear time.  **Non-wear time:** 120 epochs (30min) with zero values.  **Cut-points:** Pfeiffer *et al.* 2006 | MVPA  [min/hr]  LPA  [min/hr]  SA  [mean (SEM) mins/hr] | Age; sex; BMI; parental education, childcare centre. | Mixed model ANOVA | 93.5% |
| Lahuerta-Contell *et al.* 2021  Spain | Cross- sectional | **Sample:**  n = 116  n childcare centres = 6  **Age:**  Range: 3-4 years  Mean: 4.3 ±0.5 years  **Gender:**  49% girls. | Recess time in these ECE institutions ranged from  30 min to 45 min, took place in an outdoor environment and was a space to play freely without any educational purpose. | Structured movement session held in an indoor classroom, lasted approximately 45 min; The sessions took place once a week | **Devices:** ActiGraph GT3X+ accelerometers.  **Epoch:** 15s.  **Valid data:** 5 consecutive days.    **Non-wear time:** not reported    **Cut-points:** Van Cauwenberghe et al 2011 | MVPA [min/session]  [% ECE day MVPA] | Covariates not included. | Descriptive statistics | 85.3% |
| Raustorp *et al.* 2012  USA & Sweden | Cross -sectional | **Sample:**  n = 50  n preschools = 4  **Age:**  Range: 40-67 months  Mean: 4.3 ± 0.4 years  **Gender:**  48% girls | Researchers clocked and recorded all outdoor episodes. | Researchers clocked and recorded all indoor episodes. | **Device:** ActiGraph GT1M accelerometer  **Epoch:** 15s  **Valid data:** 4 days valid data (missing days replaced with subject’s mean scores).  **Non-wear time:** not reported  **Cut-points:** Sirard *et al*. 2005. | MVPA  [% time]  LPA  [% time]  SB  [% time]  TPA  [cpm] | Covariates not included. | Paired t-tests. | 86.2% |
| Schlechter *et al.* 2017  USA | Cross -sectional | **Sample:**  n = 73  n preschools = 2  **Age:**  Range: 3-6 years  Mean: 4.36 ± 0.85 years  **Gender:**  51.6% girls | Video recorded (Apple™ iPod Touch 5th Generation camera and a wide-angle lens) in each classroom. | 2 tripod iPods captured indoor activity within the classroom. The lead teacher used a waist-worn iPod camera. | **Device:** ActiGraph GT1M accelerometer  **Epoch:** 15s  **Valid data**: Child needed to be present in episode and segments.  **Non-wear time:** 20 consecutive minutes of zero values.  **Cut-points:** Van Cauwenberghe *et al.* 2011. | ST  [% time]  TPA  [% time] | Class, centre, subject, day-by-class interaction. | Mixed model regression. | 100% |
| Tandon *et al.*  2015  USA | Cross-sectional | **Sample:**  n = 98  n childcare centres = 10  **Age:**  Range: 3-5 years  Mean 4.5 years  **Gender:**  49% girls | Direct observation noting child location and time outdoors. | Direct observation noting child location and time indoors. | **Device:** ActiGraph GT3X accelerometer  **Epoch:** 15s  **Valid data:** not reported  **Non-wear time:** not reported  **Cut-points:** Pate *et al.* 2006 | MVPA [min/day, % per day]  LPA  [min/day, % per day]  ST  [min/day, % per day] | Age, gender, accelerometer wear time, clustering of children within childcare centres and among observations from a single child as random effects. | Mixed effects linear regressions. | Not reported |
| Tandon *et al.* 2018  USA | Cross -sectional | **Sample:**  n = 46  n childcare centres = 5  **Age:**  Range: 3-5 years  Mean: 4.5 years  **Gender**:  36% girls | QStarz GPS device. GPS and accelerometer data were combined via the Personal Activity and Location Measurement System. Indoors was differentiated to outdoors via algorithms that used satellite signal strength or signal-to-noise (SNR) ratios detected by GPS devices. | QStarz GPS device.  SNR <250 = indoor.  Direct observation for 2 days at each centre validated the GPS data regarding indoors vs. outdoor location. | **Device:** ActiGraph GT3X+ accelerometer  **Epoch:** 15s  **Valid data:** minimum of 3hr wear time.  **Non-wear time:** not reported  **Cut-points:**  Pate *et al.* 2006. | MVPA  [% time]  LPA  [% time]  ST  [% time] | Total duration, sex, weather. | Linear mixed effects model. | Not reported |
| Trost *et al.* 2008  USA | RCT | **Sample:**  n = 42  n childcare centres = 1  **Age**:  Range: 3-5 years  Mean (years): Intervention (4.1 ± 0.7); control (4.0 ± 0.7).  **Gender:**  45% girls | Free choice outdoor time directly observed using Observational System for Recording Activity in Preschoolers (OSRAP). | Free choice indoor PA using OSRAP. | **Device:** ActiGraph WAM7164 accelerometer  **Epoch:** 15s  **Valid data:** not reported  **Non-wear time:** not reported  **Cut-points:** Sirard *et al.* 2005. | MVPA  [% MVPA] | Not reported | Mixed-model repeated-measures ANOVA (PROC Mixed).  Logistic regression analyses. | 91.5% |
| Vanderloo *et al.* 2013  Canada | Cross-sectional | **Sample:**  n = 31  n childcare centres = 13  **Age:**  Range: not stated  Mean: 4.1 ± 0.85 years  **Gender:**  45% girls | The times and durations spent outdoors were recorded at each site. | The times and durations spent indoors were recorded at each site. | **Device:** ActiCal accelerometer  **Epoch:** 15s  **Valid data:** not reported  **Non-wear time:** not reported  **Cut-offs**: Pfeiffer *et al.*'s 2006. | MVPA  [min/hr]  SA  [min/hr]  TPA  [min/hr] | Not reported | Wilcoxon signed-rank tests. | Not reported |

**Table 2. Characteristics of studies comparing outdoor and indoor play**

| **Author, year, country** | **Study design** | **Participants: n, age, gender** | **Exposure description** | **Comparison description** | **Outcome assessment** | **Outcomes**  **[unit]** | **Covariates** | **Statistical analysis** | **Percentage complete data** |
| --- | --- | --- | --- | --- | --- | --- | --- | --- | --- |
| Henderson *et al.*  2015  USA | Cross-sectional | **Sample:**  n = 447  n childcare centres = 35  **Age:**  Range: 3-5 years  Mean: 4.7 ± 0.7 years  **Gender:**  50% girls | Staff participation in outdoor play and staff encouragement of PA in outdoor play recorded via environmental audit (Henderson *et al.* 2011). | Staff participation in indoor play and staff encouragement of PA in indoor play recorded via environmental audit (Henderson *et al.* 2011). | **Device:** ActiGraph GT1M accelerometer  **Epoch:** 5s  **Valid data:** not reported  **Non-wear time:**  60 consecutive minutes of zero values  **Cut-points:** Evenson *et al.* 2008 | MVPA  [adjusted mean percent of MVPA] | Not applicable | Linear mixed models. | 87.0% |
| Mazzucca *et al.* 2018  USA | Cross-sectional | **Sample:**  n = 559  n early care and education centres = 50  **Age:**  Range: 3-5 years  Mean: Not reported  **Gender:**  50% girls | Estimates of physical activity and sedentary behaviour during outdoor play were calculated using the total time observed within the EPAO. | Estimates of physical activity and sedentary behaviour during indoor play were calculated using the total time observed within the EPAO. | **Device:** ActiGraph GT1M accelerometer  **Epoch:** 15s  **Valid data:** not reported.  **Non-wear time:** 60+mins zero values.  **Cut-points:** Pate *et al.* 2006 | MVPA  [min/hr]  LPA  [min/hr]  SB  [min/hr]  TPA  [min/hr] | **Child:** age, gender.  **Centre:** monthly fees, star rating, size, portable play equipment (variety and use), summary scores (natural elements and fixed portable play equipment variety).  **Teacher:** BMI, years of experience, PA training.  **Weather:** temperature, precipitation, humidity variety. | Four one-way analysis of variances. | Not reported |
| Tandon *et al.*  2015  USA | Cross-sectional | **Sample:**  n = 98  n child care centres = 10  **Age:**  Range: 3-5 years  Mean 4.5 years  **Gender:**  49% girls | Direct observation noting child location and if they were engaging in active outdoor play. | Direct observation noting child location and if they were engaging in child-led and teacher-led active indoor play and indoor time. | **Device:** ActiGraph GT3X accelerometer  **Epoch:** 15s  **Valid data:** not reported  **Non-wear time:** not reported  **Cut-points:** Pate *et al.* 2010 | MVPA [min/day, % per day]  LPA  [min/day, % per day]  ST  [min/day, % per day] | Age, gender, accelerometer wear time, clustering of children within childcare centres and among observations from a single child as random effects. | Mixed effects linear regressions. | Not reported |

**Table 3. Characteristics of studies assessing outdoor play space**

| **Author, year, country** | **Study design** | **Participants: n, age, gender** | **Exposure description** | **Comparison description** | **Outcome assessment** | **Outcomes**  **[unit]** | **Covariates** | **Statistical analysis** | **Percentage complete data** |
| --- | --- | --- | --- | --- | --- | --- | --- | --- | --- |
| **Availability of outdoor play area** | | | | | | | | | |
| Gubbels *et al.* 2018  Netherlands | Cross sectional | **Sample:**  n = 152  n childcare centres = 22  **Age:**  Range: 1-3 years  Mean: 34.1 ± 8.97 months  **Gender:**  52.3% girls | Number of outdoor play areas. | Number of indoor active play area. | **Device:** ActiGraph GT3X accelerometer  **Epoch:** 10s  **Valid data:** Criteria by Troiano (2007) – at least 1d  **Non-wear time:** Not reported  **Cut-points:** Pate *et al.* 2006 | MVPA  [% of ECE time]  SB  [% of ECE time] | Season, parental educational level | Multiple multivariate linear regression analyses. | 71.7% |
| Olesen *et al.* 2013  Denmark | Cross-sectional | **Sample:**  n = 591  n preschools = 23  **Age:**  Range: 5-6 years  Mean: 5.8 ± 0.3 years  **Gender:**  50.5% girls | Direct observation of location of the preschool building was a measure of the number of sides of the preschool building that were accessible for the children when playing on the playground. | Direct observation of number of rooms for children to be active daily. | **Devices:** ActiGraph GT1M and GT3X accelerometers  **Epoch:** 15s  **Valid data:** 3 days with at least 3hr measurement time  **Non-wear time:** 60 consecutive mins of zero values  **Cut-points:** Evenson *et al.* 2008 | MVPA  [% of ECE time] | Gender, rainy days, preschool type, % afternoon hours, location (rural). | Pearson’s x ²; multilevel mixed modelling. | 67.90% |
| Stephens *et al.* 2014  USA | Cross-sectional | **Sample:**  n = 1,352  n childcare centres = 176  **Age**  Range: 2 years 10 months - 5 years 11 months  Mean: 3.39 years  **Gender:**  Not stated | Availability of outdoor play space, assessed through a site inventory by research staff. | Availability of indoor play space, assessed through a site inventory by research staff. | **Device:** ActiGraph GT3X accelerometer  **Epoch:** 15s  **Valid data:** 1 day wear time  **Non-wear time:** 60 consecutive minutes of zero values  **Cut-points:** Pate *et al*. 2006 | MVPA  [min/hr wear time] | **Centre-level:** Child and Adult Care Food Program (CACFP), Head Start, and NYC DPHO status; mean classroom size; operating hours; student-teacher ratio; teacher turnover; staff PA training; play space availability.  **Child-level**:  Age, sex, race/ethnicity, BMI. | 2 hierarchical linear models | 92.3% |
| Zhang *et al* 2021  Canada | Cross-sectional | **Sample:**  n = 242  n childcare centres = 19  **Age**  Range: 1.6 years - 5 years  Mean: toddlers 2.2±0.4 years; preschoolers 3.4 ±0.6 years  **Gender:**  Toddlers: 50% boys  Preschoolers: 52% boys | Functional and developmental needs of play yards assessed using the Children's Physical Environments Rating Scale | Physical activity areas in indoor space: Physical (gross motor) area, music area, and dramatic/fantasy play area assessed using the Children's Physical Environments Rating Scale | **Device:** ActiGraph WGT3XBTaccelerometer  **Epoch:** 15s  **Valid data:** at least ≥1 hr of wear time on ≥3 days  **Non-wear time:** ≥20 consecutive minutes of zero values  **Cut-points:** Pate *et al*. 2006; Trost *et al*. 2012 | MVPA  [min/hr at ECEC]  LPA [min/hr at ECEC]  ST [min/hr at ECEC] | Age, sex, mean temperature, mean precipitation, and center location | Linear mixed models | 95.7% |
| **Absolute size of outdoor play area** | | | | | | | | | |
| Bell *et al.*  2015  Australia | Cross-sectional | **Sample:**  n = 328  n childcare centres = 20  **Age:**  Range: 3-5 years  Mean: Not reported  **Gender:**  43% girls | EPAO/document review. Large outdoor play area (>400m^2^). | Small play area (≤400 m^2^). | **Devices:** Yamax SW200 and SW700 pedometers  **Epoch:** N/A  **Valid data:** Not reported  **Non-wear time:** N/A  **Cut-points:** N/A | Steps  [counts/min] | Clustering within centres. | One way ANOVA (bivariate associations); linear regression (independent associations of sig. (p < 0.05) correlates of steps. | 61.1% |
| Boldeman *et al.* 2011  Sweden and USA | Cross-sectional | **Sample:**  n = 172 (Sweden); 33 (USA)  n preschools = 9 (Sweden); 2 (USA)  **Age:**  Range: 3-5 years  Mean: not reported  **Gender:**  47.7% girls (Sweden); 51.5% (USA). | Ordinal Outdoor Play Environment Categories (OPEC) scores; Total outdoor area (scoring adapted to typical size ranges for outdoor environments of Southern Sweden and North Carolina, USA): <1200m^2^; 1200—3000m^2^; > 3000m^2^. | Reference category unclear. | **Devices:** Yamax SW200 pedometer  **Epoch:** N/A  **Valid data:** Not reported  **Non-wear time:** N/A  **Cut-points:** N/A | Steps  [counts/min ECE] | Parental education, environment category of preschool, BMI, gender, health status, attendance. | Kendall’s tau-b correlation coefficient; linear mixed model analysis. | 68.0% (Sweden)  57.0% (USA) |
| Chen et al. 2020  Sweden | Cross-sectional | **Sample:**  n = 369  n preschools = 27  **Age:**  Range: 3-5 years  Mean: 4.7 ± 0.4 years  **Gender:**  45% girls | Playground size around 900m^2^, >2700m^2^ and outdoors activity (all time at the preschool is spent outdoors). | Playground size <200m^2^ | **Device:** ActiGraph GT3X+ accelerometer  **Epoch:** 10s  **Valid data:** 10h/d wear time for 3d  **Non-wear time:** 60+mins zero values  **Cut-points :** Butte *et al*. 2014. | MVPA  [min/ECE day]  LPA  [min/ECE day]  ST  [min/ECE day]  Steps  [counts/day] | Child age, sex, BMI, and parental education. | Linear Mixed Models (LMM); random intercept at preschool level. | 91.0% |
| Dowda *et al.*  2009  USA | Cross-sectional | **Sample:**  n = 299  n preschools = 24  **Age:**  Range: 3-5 years  Mean: Not reported  **Gender:**  50% girls | Playground size was measured and averaged: all playgrounds ≥4157m^2^. | Playground size was measured and averaged: all playgrounds <4157m^2^. | **Device:** ActiGraph 7164 accelerometer  **Epoch:** 15s  **Valid data:** 5hr/day ECE attendance  **Non-wear time:** 60mins consecutive zero values  **Cut-points :** Pate *et al*. 2006. | MVPA  [min/h ECE time]  ST  [min/h ECE time] | BMI, race, gender, age, and parental education of the child, with preschool as a random variable. | Mixed-model analyses of variance. | Not reported |
| Henderson *et al.*  2015  USA | Cross-sectional | **Sample:**  n = 447  n childcare centres = 35  **Age:**  Range: 3-5 years  Mean: 4.7 ± 0.7 years  **Gender:**  50% girls | Playground area (upper quartile split) ≥5436 ft² | Playground area (upper quartile split) <5436 ft² | **Device:** ActiGraph GT1M accelerometer  **Epoch:** 5s  **Valid data:** not reported  **Non-wear time:** 60 consecutive minutes of zero values  **Cut-points :** Evenson *et al.* 2008 | MVPA  [adjusted mean percent of MVPA] | Not applicable. | Linear mixed models. | 87.0% |
| Hinkley *et al.*  2016  Australia | Cross-sectional | **Sample:**  n = 1,002  n childcare centres = 136  **Age:**  Range: 3-5 years  Mean: 4.6 ± 0.6 years  **Gender:**  46% girls | Areas measured (m^2^) | N/A | **Device:** ActiGraph GT1M accelerometer  **Epoch:** 15s  **Valid data:** Data after 4am, <18hrs/d.  **Non-wear time:** 20+ consecutive minutes of zero values  **Cut-points:** PA >100 cpm | TPA  [% time] | Total weekly hours of centre attendance was the only covariate identified for girls and controlled for in theultivariable models. | Bivariate analysis; multilevel mixed effects models. | 73.0% |
| Ng *et al.*  2020  Australia | Controlled pre-post | **Sample (control):**  n = 159 (138)  n childcare centres = 6 (5)  **Age:**  Range: 2-5 years  Mean: 2.8 ± 0.8 years  **Gender:**  51.1% girls | Modified version of the Environment and Policy Assessment and Observation (EPAO) Instrument (adapted to Australian context); ‘Total playing area’ was rated on a scale from 0 (no playing area) to 10 (very large area) by comparing all ECEC  outdoor playing areas and dividing them into 10 terciles. | Unclear. | **Device:** ActiGraph GTX3+ accelerometer  **Epoch:** 15s  **Valid data:** At least 75% wear time on 1d ECE time  **Non-wear time:** 20+ consecutive minutes of zero values  **Cut-points :** Pate *et al.* 2006 | MVPA  [min/d ECE]  TPA  [min/d ECE] | Time (baseline and follow-up), group (intervention or control), and the interaction between group and time as covariates. | Multiple multivariate linear regression analyses; stepwise deletion of independent variables. | 47.5% (int)  57.3% (con) |
| Olesen *et al.* 2013  Denmark | Cross-sectional | **Sample:**  n = 441  n preschools = 42  **Age:**  Range: 5-6 years  Mean: 5.8 ± 0.3 years  **Gender:**  50.5% girls | Area of playground (m²)  Continuous measures: 567–5175 m^2^ | N/A | **Devices:** ActiGraph GT1M and GT3X accelerometers  **Epoch:** 15s  **Valid data:** 3 days with at least 3hr measurement time  **Non-wear time:** 60 consecutive mins of zero values  **Cut-points:** Evenson *et al.* 2008 | MVPA  [% of ECE time] | Gender, rainy days, preschool type, % afternoon hours, location (rural). | Pearson’s x ²; multilevel mixed modelling. | 74.62% |
| Saunders *et al.* 2019  USA | RCT | **Sample (control):**  n = 188 (190)  n childcare centres = 16  **Age:**  Range: 4 years only  Mean: 4.5 ± 0.4 years  **Gender (control):**  51% girls (49%) | SHAPES Inventory  Assessment to measure classroom and playground size; dichotomized as the two lower terciles vs. the higher tercile,  ≥ 11,178 ft^2^. | <11,178 ft^2^ | **Devices:** ActiGraph GT1M and GT3X accelerometers  **Epoch:** 15s  **Valid data:** 3 days with at least 3hr measurement time  **Non-wear time:** 60 consecutive mins of zero values  **Cut-points:** Pate *et al.* 2006 | MVPA  [min/hr ECE day] | Baseline MVPA and wave, and centre was treated as a random variable. | ANCOVA | 100% |
| Sugiyama *et al.*  2012  Australia | Cross-sectional | **Sample:**  n = 107  n childcare centres = 10  **Age:**  Range: 3-5 years  Mean 4.1 ± 0.6 years  **Gender:**  46% girls | Outdoor play area >400m^2^; use of indoor spaces for motor activities (yes or no) | Outdoor play area <400m^2^; use of indoor spaces for motor activities (yes or no) | **Device:** ActiGraph GT1M accelerometer  **Epoch:** 15s  **Valid data:** 3 days in ECE  **Non-wear time:** not reported  **Cut-points:** Sirard *et al.* 2005 | MVPA  [min/ECE day]  ST  [min/ECE day] | Age, gender, accelerometer wear time. | Multi-level linear regression | 83.2% |
| Tonge *et al.*  2020  Australia | Cross-sectional | **Sample:**  n = 316  n ECEs = 8  **Age:**  Range: 2-5 years  Mean: not reported  **Gender:**  Not reported | Outdoor area ≥ 400m^2^ | Outdoor area < 400m^2^ | **Device:** ActiGraph GT3X+ accelerometer  **Epoch:** 15s  **Valid data:** minimum 180mins/d, and minimum 1d.  **Non-wear time:** accelerometers cleaned using a 20-mins non-wear time  **Cut-points :** Pate *et al.* 2006 | MVPA  [min/h ECE time]  ST  [min/h ECE time]  TPA  [min/h ECE time] | ECE centre, sex | Multivariate linear regression analysis. | 64.5% |

| **Density of outdoor play area** | | | | | | | | | |
| --- | --- | --- | --- | --- | --- | --- | --- | --- | --- |
| Cardon *et al.*  2008  Belgium | Cross-sectional | **Sample:**  n = 789  n preschools = 39  **Age:**  Range: 4-5 years  Mean: 5.3 ± 0.4 years  **Gender:**  47% girls | The researchers measured all playgrounds to determine the  play space per child (children per m^2^). | N/A | **Device:** Yamax SW-200 pedometer  **Epoch:** N/A  **Valid data:** not reported  **Non-wear time:** N/A  **Cut-points:** N/A | Steps  [counts/min] | Single predictor  two-level (school-pupil) model was used. To test the  significance of the variance at the school level Z-scores were calculated. | Not reported | 97% |
| Gubbels *et al.* 2018  Netherlands | Cross- sectional | **Sample:**  n = 152  n childcare centres = 22  **Age:**  Range: 1-3 years  Mean: 34.1 ± 8.97 months  **Gender:**  52.3% girls | Relative size of outdoor play areas (m^2^ per child) | The number of indoor areas in which children participated in physically active play was summed | **Device:** ActiGraph GT3X accelerometer  **Epoch:** 10s  **Valid data:** 7d wear-time with at least one valid childcare day  **Non-wear time:** not reported  **Cut-points:** Pate *et al.* 2006 | MVPA  [% time at ECE]  ST  [% time at ECE] | Season, parental educational level | Multiple multivariate linear regression analyses. | 71.7% |
| Lahuerta-Contell *et al.* 2021  Spain | Cross- sectional | **Sample:**  n = 116  n childcare centres = 6  **Age:**  Range: 3-4 years  Mean: 4.3 ± 0.5 years  **Gender:**  49% girls | Children per m^2^ assessed based on playground area obtained from Google Earth into geographic information  system software (ArcGIS 10.2) | Researchers measured the classroom | **Devices:** ActiGraph GT3X+ accelerometers.  **Epoch:** 15s.  **Valid data:** 5 consecutive days.    **Non-wear time:** not reported    **Cut-points :** Van Cauwenberghe et al 2011 | VPA [min/h]  MVPA [min/h]  LPA [min/h]  ST [min/h] | Participant clustering in class groups and school groups | Mixed-effect regression analysis | 85.3% |
| Van Cauwenberghe *et al.*  2012  Belgium | Before-after | **Sample:**  n = 128  n preschools = 4  **Age:**  Range: 4-6 years  Mean: 5.1 ± 0.6 years  **Gender:**  46% girls | Available outdoor play space during intervention: 16.7 m^2^ per child | Available outdoor play space at baseline: 7.4 m^2^ per child | **Device:** ActiGraph GT1M accelerometer  **Epoch:** 15s  **Valid data:** not reported  **Non-wear time:** not reported  **Cut-points:** Van Cauwenberghe *et al.* 2011 | MVPA  [mins]  LMVPA  [mins]  ST  [mins] | Child gender, age, accelerometer wear time | Two-level linear regression | 84% |
| Vega-Perona *et al* 2022  Spain | Cross-sectional | **Sample:**  n = 120  n childcare centres = 7  **Age:**  Range: 2-3 years  Mean: 2.5 ± 0.5 years  **Gender:**  46% girls | Google Earth Pro (GEP) software was used to provide an estimate of the playground spatial area (m2) and the polygon measurement tool. Average playground density (children/m^2^) was calculated by dividing the number of children in the playground by the playground size available for use outdoor time. | Indoor classroom and indoor playground area (m2) were measured manually with a meter by researchers, children/m^2^ calculated by dividing the  number of toddlers in the classroom by the classroom size available for use indoor time. | **Devices:** ActiGraph GT3X+ accelerometers  **Epoch:** 15s  **Valid data:** 5 consecutive days    **Non-wear time:** naptime    **Cut-points :** Trost *et al* 2012 | TPA [mins]  MVPA  [mins]  LPA  [mins]  ST  [mins] | Age, gender, BMI | Mixed-effects linear models | 67% |

**Table 4. Characteristics of studies assessing the use of outdoor space external to ECEC premises**

| **Author, year, country** | **Study design** | **Participants: n, age, gender** | **Exposure description** | **Comparison description** | **Outcome assessment** | **Outcomes**  **[unit]** | **Covariates** | **Statistical analysis** | **Percentage complete data** |
| --- | --- | --- | --- | --- | --- | --- | --- | --- | --- |
| **Outdoor Environment** | | | | | | | | | |
| Barbosa *et al.*  2016  Brazil | Cross-sectional | **Sample:**  n = 370  n preschools = 8  **Age:**  Range = 4-6 years  Mean = 5.2 ± 0.8 years  **Gender:**  49.6% girls | Availability of outdoor space (park) near ECEC assessed via questionnaire completed by school principals. | Availability of indoor recreation room; no availability of outdoor space (park). | **Device:** ActiGraph GT3X accelerometer  **Epoch:** 1s  **Valid data:** 3d data for minimum of 360 mins (4-5 years) or 120 mins (6 years)  **Non-wear time:** not reported  **Cut-points:** 4-5 year old Sirard et al. 2005; 6 year old Van Cauwenberghe et al | SB  [min/h]  TPA  [% children in 75^th^ PA percentile] | Environmental variables, infrastructure, gender, BMI, age, school region. | Chi-square test. Binary logistic regression analysis. | 92.3% |
| Määttä *et al.*  2019  Finland | Cross-sectional | **Sample:**  n = 778  n preschools = 66  **Age:**  Range: 3-6 years  Mean: 4.7 years  **Gender:**  Not reported | Frequency of nature trips and frequency of visits to play parks. | Frequency of visits to gym or other indoor facility. | **Device:** ActiGraph W-GT3X accelerometer  **Epoch:** 15s  **Valid data:** Attended preschool 2d/wk, minimum wear 240min in preschool hours.  **Non-wear time:** 10 mins zero values  **Cut-points:** Evenson *et al.* 2008 | TPA  [min/hour]  SB  [min/hour] | Age, gender, average attendance at preschool, study season | Multilevel linear regression models. | 95.0% |

**Table 5. Characteristics of studies investigating outdoor play equipment**

| **Author, year, country** | **Study design** | **Participants: n, age, gender** | **Exposure description** | **Comparison description** | **Outcome assessment** | **Outcomes**  **[unit]** | **Covariates** | **Statistical analysis** | **Percentage complete data** |
| --- | --- | --- | --- | --- | --- | --- | --- | --- | --- |
| **Portable Outdoor Play Equipment** | | | | | | | | | |
| Copeland *et al.* 2016  USA | Cross- sectional | **Sample:**  n = 377  n childcare centres = 29  **Age:**  Range: 3-6 years  Mean: 4.3 ± 0.7 years  **Gender:**  51% girls. | Three measures of portable play equipment for outdoor sessions: number of balls and pieces of riding equipment, and number of types of portable play equipment from a list of 15. | Three measures of portable play equipment for indoor sessions: number of balls and pieces of riding equipment, and number of types of portable play equipment from a list of 15. | **Device:** ActiCal uniaxial accelerometer  **Epoch:** 15s    **Valid data:** 24 hours excluding sleep and non-wear time.  **Non-wear time:** 120 epochs (30min) with zero values.  **Cut-points :** Pfeiffer *et al.* 2006 | MVPA  [min/hr] | Age; sex; BMI; parental education, childcare centre. | Mixed model ANOVA | 93.5% |
| Dowda *et al.*  2009  USA | Cross-sectional | **Sample:**  n = 299  n preschools = 24  **Age:**  Range: 3-5 years  Mean: Not reported  **Gender:**  50% girls | Counts were made of portable equipment  (eg, balls and tricycles) brought to the playground. Preschools were divided into 2 groups on the basis of the median values for portable equipment (range: 0–8 pieces; median: 1 piece) | N/A | **Device:** ActiGraph 7164 accelerometer  **Epoch:** 15s  **Valid data:** 5hr/day ECE attendance  **Non-wear time:** 60mins consecutive zero values  **Cut-points :** Pate *et al*. 2006. | MVPA  [min/h ECE time]  ST  [min/h ECE time] | BMI, race, gender, age, and parental education of the child, with preschool as a random variable. | Mixed-model analyses of variance. | Not reported |
| Gubbels *et al.* 2018  Netherlands | Cross- sectional | **Sample:**  n = 152  n childcare centres = 22  **Age:**  Range: 1-3 years  Mean: 2.84 ± 0.75 years  **Gender:**  52.3% girls | Amount of portable outdoor play equipment rated as present or not: balls, portable climbing structures, floor play equipment (e.g., tumbling mats), jumping play equipment (e.g., bouncing balls), push/pull toys (e.g., doll wagon), riding toys (e.g., tricycles), rocking or twisting toys (e.g., rocking horse), sand/water tables, sand/water play toys (e.g., scoops), portable slides, small portable pools, portable tunnels | Amount of portable indoor equipment rated as present or not. | **Device:** ActiGraph GT3X accelerometer  **Epoch:** 10s  **Valid data:** 7d wear-time with at least one valid childcare day  **Non-wear time:** not reported  **Cut-points:** Pate *et al.* 2006 | MVPA  [% weartime at ECE]  ST  [% weartime at ECE] | Season, parental educational level | Multiple multivariate linear regression analyses. | 71.7% |
| Hannon and Brown  2008  USA | Pre-post | **Sample:**  n = 64  n preschools = 1  **Age:**  Range 3-5 years  Mean: not reported  **Gender:**  53% girls | Recorded time spent playing outdoors after the introduction of portable activity-friendly play equipment (5 days). Observational System for Recording Physical Activity in Children – Preschool Version (OSRAC-P). | Recorded time spent playing outdoors before the introduction of portable activity-friendly play equipment (5 days). Observational System for Recording Physical Activity in Children – Preschool Version (OSRAC-P). | **Device:** ActiGraph GT1M accelerometer  **Epoch:** 15s  **Valid data:** Not reported  **Non-wear time:** Not reported  **Cut-points:** Sirard *et al.* 2005 | MVPA [%outdoor playtime]  LPA  [% outdoor playtime]  SB  [%outdoor playtime]  VPA  [%outdoor playtime] | Gender, age, intervention effect | ­­Repeated measures compared preschoolers’ PA levels before and after the introduction of new play equipment. | 84.2% |
| Määttä *et al.*  2019  Finland | Cross-sectional | **Sample:**  n = 778  n preschools = 66  **Age:**  Range: 3-6 years  Mean: 4.7 years  **Gender:**  Not reported | Total amount of portable equipment in the playground: Balls, Skipping ropes, Balance equipment, Riding toys, Sticks, Goals, Sleds, Snow pushers | N/A | **Device:** ActiGraph W-GT3X accelerometer  **Epoch:** 15s  **Valid data:** Attended preschool 2d/wk, minimum wear 240min in preschool hours.  **Non-wear time:** 10 mins zero values  **Cut-points:** Evenson *et al.* 2008 | TPA  [min/hour] | Child’s age and gender, municipality, and season of measurement and clustered with preschool group | Multilevel linear regression models. | 95.0% |
| Ng *et al.*  2020  Australia | Controlled pre-post | **Sample (control):**  n = 159 (138)  n childcare centres = 6 (5)  **Age:**  Range: 2-5 years  Mean: 2.8 ± 0.8 years  **Gender:**  51.1% girls | Availability of nine items: balls,  climbing structures (e.g., ladders), floor play equipment (e.g., tumbling mats), jumping equipment  (e.g., jump ropes, hula hoops), push/pull toys (e.g., wagons), riding toys (e.g., tricycles, cars), slides,  sand/water toys (e.g., buckets, scoops), and twirling equipment (e.g., ribbons, batons). Items were coded 1 if present and 0 if not | N/A | **Device:** ActiGraph GTX3+ accelerometer  **Epoch:** 15s  **Valid data:** At least 75% wear time on 1d ECE time  **Non-wear time:** 20+ consecutive minutes of zero values  **Cut-points :** Pate *et al.* 2006 | MVPA  [min/d ECE]  TPA  [min/d ECE] | Time (baseline and follow-up), group (intervention or control), and the interaction between group and time as covariates. | Multiple multivariate linear regression analyses; stepwise deletion of independent variables. | 47.5% (int)  57.3% (con) |
| Olesen *et al.* 2013  Denmark | Cross-sectional | **Sample:**  n = 441  n preschools = 42  **Age:**  Range: 5-6 years  Mean: 5.8 ± 0.3 years  **Gender:**  50.5% girls | On the playground the number of accessible portable (eg, balls) play opportunities per child was counted | N/A | **Devices:** ActiGraph GT1M and GT3X accelerometers  **Epoch:** 15s  **Valid data:** 3 days with at least 3hr measurement time  **Non-wear time:** 60 consecutive mins of zero values  **Cut-points:** Evenson *et al.* 2008 | MVPA  [% of ECE time] | Gender, rainy days, preschool type, % afternoon hours, location (rural). | Pearson’s x ² | 74.62% |
| **Fixed Outdoor Play Equipment** | | | | | | | | | |
| Copeland *et al.* 2016  USA | Cross- sectional | **Sample:**  n = 377  n childcare centres = 29  **Age:**  Range: 3-6 years  Mean: 4.3 ± 0.7 years  **Gender:**  51% girls. | < 9 pieces of fixed equipment: climber, slide, swings, tire swing, monkey bars, climbing ropes/chains, tunnels, sandbox, other digging area, water/sensory  table, place to sit/quiet activities, dramatic play fixed structure (e.g., playhouse), basketball hoop or other aiming structures, climbing wall, fixed  balance beam, fixed rocking/twisting toys, fixed easels, and a storage sheds | N/A | **Device:** ActiCal uniaxial accelerometer  **Epoch:** 15s    **Valid data:** 24 hours excluding sleep and non-wear time.  **Non-wear time:** 120 epochs (30min) with zero values.  **Cut-points :** Pfeiffer *et al.* 2006 | MVPA  [min/hr] | Age; sex; BMI; parental education, childcare centre. | Mixed model ANOVA | 93.5% |
| Dowda *et al.*  2009  USA | Cross-sectional | **Sample:**  n = 299  n preschools = 24  **Age:**  Range: 3-5 years  Mean: Not reported  **Gender:**  50% girls | Counts were made of fixed playground equipment for physical activity (eg, jungle gyms, slides, and swings). Preschools were divided into 2 groups on the basis of the  median values for  fixed equipment (range: 3–14 pieces; median: 8 pieces). | N/A | **Device:** ActiGraph 7164 accelerometer  **Epoch:** 15s  **Valid data:** 5hr/day ECE attendance  **Non-wear time:** 60mins consecutive zero values  **Cut-points :** Pate *et al*. 2006. | MVPA  [min/h ECE time]  ST  [min/h ECE time] | BMI, race, gender, age, and parental education of the child, with preschool as a random variable. | Mixed-model analyses of variance. | Not reported |
| Gubbels *et al.* 2018  Netherlands | Cross- sectional | **Sample:**  n = 152  n childcare centres = 22  **Age:**  Range: 1-3 years  Mean: 2.84 ± 0.75 years  **Gender:**  52.3% girls | Amount of fixed outdoor play equipment rated as present or not: balancing surfaces (e.g., balance beams), basketball hoop or soccer goal, fixed climbing structures, merry-go-round, fixed place to play with water, sandbox, see-saw, fixed slides,  swinging equipment (e.g., swings), tricycle track or paved areas, fixed tunnels, benches, picnic tables, small stage or raised deck, play house, floor markings (e.g., colors, tracks) | Amount of fixed indoor play equipment rated as present or not | **Device:** ActiGraph GT3X accelerometer  **Epoch:** 10s  **Valid data:** 7d wear-time with at least one valid childcare day  **Non-wear time:** not reported  **Cut-points:** Pate *et al.* 2006 | MVPA  [% weartime at ECE]  SB  [% weartime at ECE] | Season, parental educational level | Multiple multivariate linear regression analyses. | 71.7% |
| Määttä *et al.*  2019  Finland | Cross-sectional | **Sample:**  n = 778  n preschools = 66  **Age:**  Range: 3-6 years  Mean: 4.7 years  **Gender:**  Not reported | Total amount of fixed equipment in the preschool yard: Sandbox, Playhouse, Swings, Spring swings, Seesaw, Climbing frames,  Slides, Balancing equipment, Merry-go-rounds | Total amount of fixed equipment indoors: Soft area for playing, Stall bars, Climbing wall or other climbing place, Floor marks, Pool/Water play equipment | **Device:** ActiGraph W-GT3X accelerometer  **Epoch:** 15s  **Valid data:** Attended preschool 2d/wk, minimum wear 240min in preschool hours.  **Non-wear time:** 10 mins zero values  **Cut-points:** Evenson *et al.* 2008 | TPA  [min/hour] | Child’s age and gender, municipality, and season of measurement and clustered with preschool group | Multilevel linear regression models. | 95.0% |
| Ng *et al.*  2020  Australia | Controlled pre-post | **Sample (control):**  n = 159 (138)  n childcare centres = 6 (5)  **Age:**  Range: 2-5 years  Mean: 2.8 ± 0.8 years  **Gender:**  51.1% girls | Availability of eight types of equipment:  structured tracks (e.g., playground markings), climbing structures (e.g., jungle gyms), see-saws, slides,  tunnels, balancing surfaces (e.g., balance beams), sandboxes, and swinging equipment (e.g., swings, ropes). Items were coded 1 if present and 0 if not; total score for ‘Fixed play equipment’ was calculated as the sum, divided by eight (number of items) and multiplied by  10 (to obtain a score from 0 to 10, higher score indicated more activity opportunities) | N/A | **Device:** ActiGraph GTX3+ accelerometer  **Epoch:** 15s  **Valid data:** At least 75% wear time on 1d ECE time  **Non-wear time:** 20+ consecutive minutes of zero values  **Cut-points :** Pate *et al.* 2006 | MVPA  [min/d ECE]  TPA  [min/d ECE] | Time (baseline and follow-up), group (intervention or control), and the interaction between group and time as covariates. | Multiple multivariate linear regression analyses; stepwise deletion of independent variables. | 47.5% (int)  57.3% (con) |
| Olesen *et al.* 2013  Denmark | Cross-sectional | **Sample:**  n = 441  n preschools = 42  **Age:**  Range: 5-6 years  Mean: 5.8 ± 0.3 years  **Gender:**  50.5% girls | On the playground the number of accessible fixed (eg, playhouse) play opportunities per child was counted | N/A | **Devices:** ActiGraph GT1M and GT3X accelerometers  **Epoch:** 15s  **Valid data:** 3 days with at least 3hr measurement time  **Non-wear time:** 60 consecutive mins of zero values  **Cut-points:** Evenson *et al.* 2008 | MVPA  [% of ECE time] | Gender, rainy days, preschool type, % afternoon hours, location (rural). | Pearson’s x ² | 74.62% |
| Sugiyama *et al.*  2012  Australia | Cross-sectional | **Sample:**  n = 107  n childcare centres = 10  **Age:**  Range: 3-5 years  Mean 4.1 ± 0.6 years  **Gender:**  46% girls | Observation by two research staff members: fixed play equipment (number) in outdoor play areas | N/a | **Device:** ActiGraph GT1M accelerometer  **Epoch:** 15s  **Valid data:** 3 days in ECE  **Non-wear time:** not reported  **Cut-points:** Sirard *et al.* 2005 | MVPA  [min/ECE day]  SB  [min/ECE day] | Age, gender, outdoor time. | Multi-level linear regression | 83.2% |

**Table 6.** **Accelerometer cut-points used in included studies**

| **Cut-points** | **Studies** | **Total (n = )** |
| --- | --- | --- |
| Butte *et al.*, 2014 | Andersen *et al.*, 2017; Chen *et al.*, 2014 | 2 |
| Evenson *et al.* 2008 | Henderson *et al.*, 2015; Määttä *et al.*, 2019; Olesen *et al.*, 2013 | 3 |
| Pate *et al.*, 2006 | Dowda *et al.*, 2006; Gubbels *et al.*, 2018; Mazzucca *et al.*, 2018; Ng *et al.*, 2020; Saunders *et al.*, 2019; Stephens *et al.*, 2014; Tandon *et al.*, 2018; Tonge *et al.*, 2020; Vanderloo *et al.*, 2013; Zhang *et al* 2021 | 10 |
| Pate *et al.*, 2010 | Tandon *et al.*, 2015 | 1 |
| Pfeiffer *et al.*, 2006 | Copeland *et al.*, 2016 | 1 |
| Sirard *et al.*, 2005 | Barbosa *et al.*, 2016; Hannon and Brown, 2008; Raustorp *et al.*, 2012; Sugiyama *et al.*, 2012; Trost *et al.*, 2008 | 5 |
| Trost *et al*., 2012 | Zhang *et al*. 2021; Vega-Perona *et al*. 2022 | 2 |
| van Cauwenberghe *et al.* 2011 | Schlechter *et al.* 2017; van Cauwenberghe *et al.*, 2011; Lahuerta-Contell et al 2021 | 3 |
